# High‐Density Type I Collagen Promotes IFN‐γ^+^ CD8^+^ T Cell Exhaustion via SAT1‐ASS1‐Mediated Glutamine Accumulation and Ferroptosis in Triple‐Negative Breast Cancer

**DOI:** 10.1002/advs.77879

**Published:** 2026-09-27

**Authors:** Jinyan Wang, Shuangyue Pan, Lu Bai, Bin Li, Tiantian Liu, Jianing Cao, Mengdi Yang, Heda Zhang, Duancheng Guo, Wenxiang Zhi, Zhonghua Tao, Xichun Hu

**Affiliations:** ^1^ Department of Medical Oncology Shanghai Pulmonary Hospital Tongji University School of Medicine Shanghai China; ^2^ Department of Medical Oncology Fudan University Shanghai Cancer Center Shanghai China; ^3^ Department of Oncology Shanghai Medical College of Fudan University Shanghai China; ^4^ Department of Ultrasonography Fudan University Shanghai Cancer Center Shanghai China; ^5^ Department of General Surgery the First Affiliated Hospital of Nanjing Medical University Jiangsu China; ^6^ Cancer Institute Fudan University Shanghai Cancer Center Shanghai China

**Keywords:** ferroptosis, glutamine, SAT1, TNBC, Type I collagen density

## Abstract

Triple‐negative breast cancer (TNBC) is an aggressive subtype characterized by poor prognosis because of a lack of effective therapeutic agents. Changes in extracellular matrix (ECM) composition, particularly type I collagen density, significantly influence crucial cellular activities. However, the association between type I collagen density and TNBC progression remains unclear. This study finds that type I collagen density and mechanical characteristics of lesions predict the pathological grade and malignant progression of TNBC patients. The results of transcriptomics and metabolomics studies suggest that high‐density type I collagen promotes TNBC ferroptosis. Further in vitro experiments indicate that high‐density type I collagen promotes ferroptosis in TNBC cells through spermidine/spermine N1‐acetyltransferase 1 (SAT1)/argininosuccinate synthase (ASS1)‐induced glutamine (Gln) accumulation. Moreover, in vitro co‐culture and in vivo experiments reveal that high‐density type I collagen increases the uptake of Gln in TNBC cells, leading to Gln deprivation of IFN‐γ^+^ CD8^+^ T cells in the tumor microenvironment, resulting in higher level of immunosuppression. Combination of ferrostatin 1 and anti‐PD‐1 therapy reverses high‐density collagen‐induced tumor progression of TNBC. The clinical samples further verify the role of collagen density in TNBC. This study reveals that high‐density type I collagen participates in TNBC progression partly through immunosuppression induced by Gln accumulation‐related ferroptosis.

## Graphical abstract

High‐density type I collagen promotes glutamine (Gln) accumulation–related ferroptosis of TNBC cells via the spermidine/spermine N1‐acetyltransferase 1 (SAT1)‐ argininosuccinate synthase (ASS1) signaling pathway, thus depriving immune cells of Gln supply and inducing IFN‐γ^+^ CD8^+^ T cell exhaustion in the TME. Combination of ferrostatin 1 and anti‐PD‐1 therapy reverses collagen density‐induced tumor progression of TNBC.

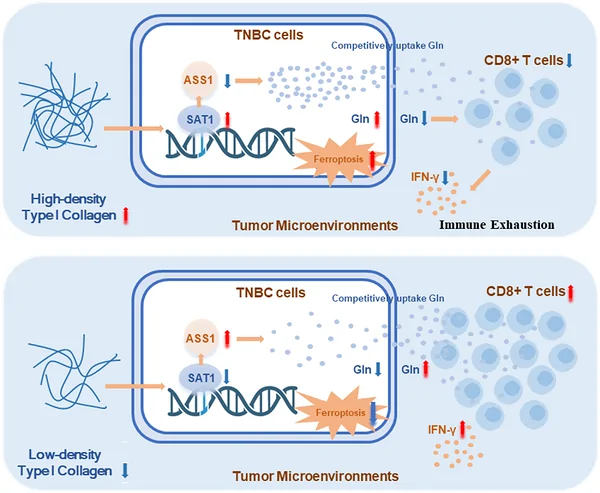

## 1 Introduction

According to Global Cancer Statistics (2022), breast cancer is the most prevalent malignancy in women, with 9.7 million new cases and 4.3 million deaths worldwide [1]. Triple‐negative breast cancer (TNBC) is a subtype of breast cancer categorized by negative immunohistochemistry (IHC) expression of the estrogen receptor (ER), progesterone receptor (PR), and human epidermal growth factor receptor 2 (Her2), accounting for approximately 15% of all breast cancer [2]. TNBC has a high recurrence and metastasis rate, as well as high‐grade and poor prognosis [3]. Therefore, understanding the underlying mechanisms of TNBC tumorigenesis and progression is crucial for developing potential treatment strategies.

It is well‐accepted that the mechanical properties of the extracellular matrix (ECM) greatly influence crucial cellular activities, including transcription, proliferation, and migration through various mechanotransduction pathways [4, 5]. In the late 1990s, Pelham et al. [6]. found that substrate stiffness influences cell‐ECM adhesion, spreading, and migration using polyacrylamide hydrogels as cell culture substrates. Since then, researchers have applied polyacrylamide gels to investigate the role of ECM stiffness in modulating cell functions, mechanotransduction, and disease progression [7, 8, 9]. However, recent studies reveal that tissues and ECMs are not linearly elastic materials but exhibit far more complex mechanical behaviors such as viscoelasticity and viscoplasticity. For example, enhanced viscoelasticity promotes hepatocellular carcinoma cell proliferation and invasion via an integrin‐β1‐tensin‐1‐YAP1 mechanotransduction pathway [10], and that ECM viscoelasticity modulates T cell function through the activator protein‐1 signaling pathway [11].

Type I collagen is the predominant structural component of ECM and provides essential architectural support for tissue integrity [12]. The increased deposition and altered organization of type I collagen – commonly referred to as desmoplasia or tumor‐associated stromal reaction – represent pathological hallmarks frequently associated with aggressive disease behavior and unfavorable clinical outcomes across multiple cancer types, including breast, pancreatic, and hepatocellular carcinomas [13, 14, 15]. Functionally, type I collagen exerts its biological influence through diverse mechanisms. As a physical scaffold, dense collagen networks generate elevated tissue stiffness and solid stress, which not only promote malignant phenotypes such as proliferation, invasion, and epithelial‐mesenchymal transition (EMT) via mechanotransduction pathways (e.g., YAP/TAZ and RhoA‐ROCK signaling), but also impair vascular perfusion and therapeutic penetration by compressing tumor vasculature [16, 17, 18]. Beyond its mechanical roles, type I collagen actively modulates cellular signaling through engagement with specific receptors, including integrins (α1β1, α2β1) and discoidin domain receptors (DDRs), thereby activating downstream survival and motility programs [19, 20, 21]. Furthermore, emerging evidence indicates that collagen‐dense matrices function as immunoregulatory barriers that restrict cytotoxic immune cell infiltration and spatially compartmentalize the tumor microenvironment (TME) [22, 23]. Recent studies have also revealed that ECM collagen can influence the metabolic programming of both tumor and stromal cells, altering nutrient availability and energetic states within the TME [24, 25]. In breast cancer specifically, tumor‐associated collagen signatures (TACS) are correlated with tumor grade, metastatic potential, and patient survival [26, 27]. Collectively, these findings establish type I collagen not merely as a passive structural element, but as an active participant in tumor progression through integrated mechanical, signaling, immunological, and metabolic mechanisms.

However, the association between type I collagen density and TNBC progression remains unclear. In this study, we discovered that high‐density type I collagen induced Gln accumulation‐related ferroptosis of TNBC cells via the SAT1‐ASS1 signaling pathway. This process deprived immune cells of Gln supply and induced IFN‐γ^+^ CD8^+^ T cell exhaustion in the TME. In addition, the combination of ferrostatin 1 (Fer‐1) and anti‐programmed cell death protein 1 (anti‐PD‐1) therapy reversed high‐density collagen‐induced tumor progression of TNBC.

## 2 Results

### 2.1 Clinical Significance of Biomechanical Properties and Type I Collagen Density in TNBC

**FIGURE 1:**
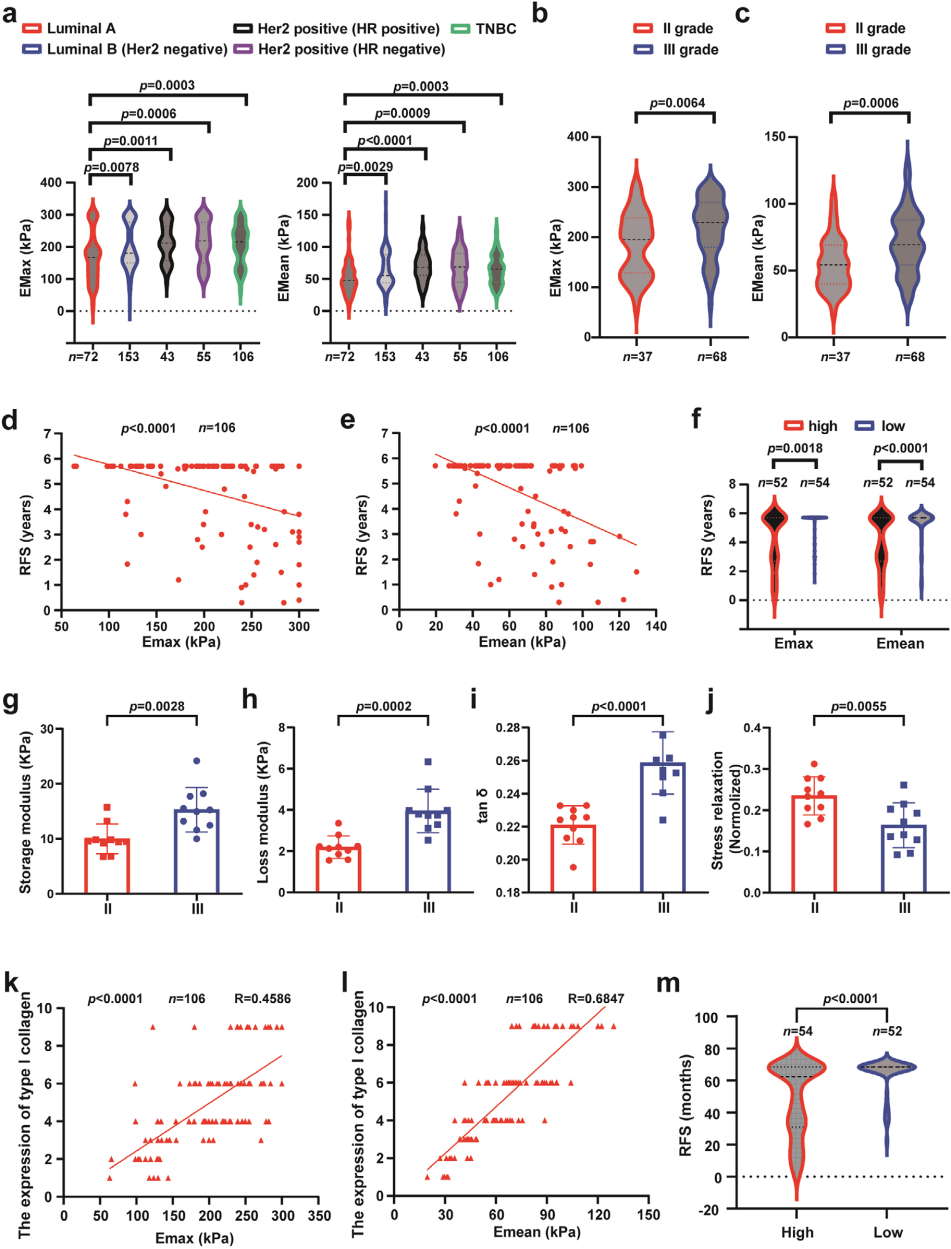
Clinical significance of biomechanical properties and type I collagen density in TNBC. (a) Differential analyses of Emax and Emean values in five breast cancer subtypes. (b,c) Differential analyses of Emax and Emean values in pathological grading of TNBC. (d,e) Scatter plots of Emax and Emean values distribution and RFS in patients with TNBC. Data are presented as a scatter plot with a linear regression line. (f) Differential analyses of RFS between high and low Emax/Emean values in patients with TNBC. (g) Differential analyses of storage modulus between II and III grade TNBC. *n* = 10. (h) Differential analyses of loss modulus between II and III grade TNBC. *n* = 10. (i) Differential analyses of tanδ between II and III grade TNBC. *n* = 10. (j) Differential analyses of stress relaxation between II and III grade TNBC. *n* = 10. (k,l) Scatter plots of Emax and Emean values distribution and the expression of type I collagen in patients with TNBC. Data are presented as a scatter plot with a linear regression line. (m) Differential analyses of RFS between high and low expression of type I collagen in patients with TNBC. Statistical significance was determined by two‐tailed unpaired t‐test (a–c,f–j,m) and Pearson's r (d,e,k,l). Violin plots showing data distribution (a–c,f,m). Data are presented as mean ± SD (g–j).

The elasticity of lesions in 428 female patients with breast cancer was examined using shear wave elastography (SWE), a noninvasive method for real‐time evaluation of soft tissue elasticity [28, 29]. Chi‐squared tests were used to investigate the associations between lesion elasticity and patients′ clinicopathologic features, including age, T stage, pathological grade, lymph node metastasis, and Ki67 expression. The results indicated that both pathological grade and Ki67 expression were closely related to maximum elastic modulus (Emax) and average elastic modulus (Emean) values of the lesions (Table 1). Patients were further classified into five subtypes according to the American Society of Clinical Oncology/College of American Pathologists guidelines, including Her2 positive (HR‐positive), Her2 positive (HR‐negative), Luminal A, Luminal B (Her2‐negative), and TNBC [30, 31]. Differential analysis revealed that luminal A breast cancer, which has a better prognosis than other subtypes [32], exhibited lower Emax and Emean values than the other four types (Figure 1a). Furthermore, chi‐squared tests among the five subtypes indicated that Emax and Emean values were specifically related to the pathological grade of TNBC (Table 2) but not in the other subtypes (Table S1). Higher pathological grades were associated with higher Emax and Emean values in TNBC (Figure 1b,c). As illustrated in the scatter diagrams, when the Emax and Emean values increased, patients′ relapse‐free survival (RFS) worsened (Figure 1d,e). Differential analysis of RFS further confirmed that higher Emax and Emean values were associated with shorter survival times (Figure 1f). Rheometry, another important method for evaluating the biomechanical properties of tissues [33], was also performed on 20 human TNBC tissue samples. Rheometric analysis revealed that higher pathological grades were associated with higher storage modulus (G′), loss modulus (G″), and tan δ, and faster stress relaxation (Figure 1g–j).

**TABLE 1:** The associations between Emax/Emean values and clinicopathologic features of breast cancer patients.

		Total	Emax (kPa)				Emean (kPa)			
Factors		*n* = 428	Low (*n* = 214)	High (*n* = 214)	*χ2*	*p*	Low (*n* = 214)	High (*n* = 214)	*χ2*	*p*
Age	<50	181	99	82	2.767	ns	96	85	1.158	ns
	≧50	247	115	132			118	129		
T stage	T1	202	113	89	5.491	ns	110	92	3.046	ns
	T2	209	94	115			96	113		
	T3	17	7	10			8	9		
Pathological grade	I	9	8	1	17.43	0.0002	7	2	16.74	0.0002
	II	237	134	103			136	101		
	III	182	72	110			71	111		
Lymph node metastasis	0	228	117	110	0.4596	ns	120	108	1.352	ns
	≧1	200	97	104			94	106		
Ki67	<30	164	102	60	17.52	<0.0001	100	62	14.34	0.0002
	≧30	266	112	154			114	152		

**TABLE 2:** The associations between Emax/Emean values and clinicopathologic features of TNBC patients.

		Total	Emax (kPa)				Emean (kPa)			
Factors		*n* = 106	Low *n* = 53	High *n* = 53	*χ2*	*p*	Low *n* = 53	High *n* = 53	*χ2*	*p*
Age	<50	47	25	22	0.4909	ns	23	24	0.03823	ns
	≧50	59	28	31			30	29		
T stage	T1	47	28	19	3.109	ns	26	21	1.028	ns
	T2	54	23	31			25	29		
	T3	5	2	3			2	3		
Pathological grade	I	1	1	0	6.388	0.041	1	0	8.45	0.0146
	II	37	24	13			25	12		
	III	68	28	40			27	41		
Lymph node metastasis	0	66	34	32	0.1606	ns	37	29	2.57	ns
	≧1	40	19	21			16	24		
Ki67	<30	7	6	1	3.824	ns	7	1	3.824	ns
	≧30	99	47	52			47	52		

Given that type I collagen constitutes the predominant structural component of the ECM and provides essential architectural support for tissue integrity [34], we next evaluated type I collagen expression via IHC in tumor lesions from 106 TNBC patients. Chi‐squared analysis demonstrated that Emax and Emean values were significantly associated with type I collagen protein levels (Table 3), with higher collagen expression consistently accompanied by elevated Emax and Emean values (Figure 1k,l). Patients with elevated type I collagen levels exhibited significantly shorter RFS (Figure 1m), indicating a strong correlation between high collagen density and unfavorable prognosis in TNBC. Collectively, these clinical findings reveal that mechanical alterations in TNBC tissues are associated with variable type I collagen expression, and elevated type I collagen expression might be a significant predictor of worsened RFS in patients with TNBC. These results support the hypothesis that biomechanical characteristics of lesions, driven by collagen density, may predict the pathological grade and malignant progression of TNBC.

**TABLE 3:** Association of Emax/Emean values with type I collagen expression in TNBC.

		Total	The expression of type I collagen (IHC)			
Factor		*n* = 106	High	Low	χ2	*p*
Emax (kPa)	High	53	40	13	5.907	0.0151
	Low	53	28	25		
Emean (kPa)	High	53	45	8	48.92	<0.0001
	Low	53	9	44		

### 2.2 Ferroptosis Is Coupled to High‐Density Type I Collagen

To investigate the role of type I collagen density in TNBC, an in vitro collagen density model was established by covering culture dishes with different concentrations of type I collagen solution [11]. This model is used to vary collagen density, which concurrently alters matrix stiffness, viscoelasticity, ligand density, and pore architecture. In the process of hydrogel formation and gelation kinetics measurement, a high concentration of type I collagen (2.5 or 1 mg mL^−1^) exhibited higher G′, G″, and tan δ, and faster stress relaxation, suggesting strong viscoelasticity and increased stiffness (2.5 mg mL^−1^, high density, G′ = 815.876 ± 11.798 Pa, G″ = 102.236 ± 1.893 Pa, tan δ = 0.207 ± 0.002, normalized stress relaxation = 0.479 ± 0.078; 1 mg mL ^−1^, modest density, G′ = 27.574 ± 0.434 Pa, G″ = 3.782 ± 0.074 Pa, tan δ = 0.180 ± 0.002, normalized stress relaxation = 0.759 ± 0.013), compared with that of the low concentration (50 µg mL ^−1^, low density, G′ = 0.312±0.006 Pa, G″ = 0.054±0.002 Pa, tan δ = 0.172 ± 0.004, normalized stress relaxation = 0.855 ± 0.024) (Figure S1a–f). In the examination of the mechanical stability, different concentrations demonstrated good mechanical stability under a gradually increasing oscillation frequency, with relatively stable values of G′ and G″ (Figure S1g–i). Consequently, the in vitro collagen density model established in this study functioned well for investigating the biological effects of varied collagen density.

**FIGURE 2:**
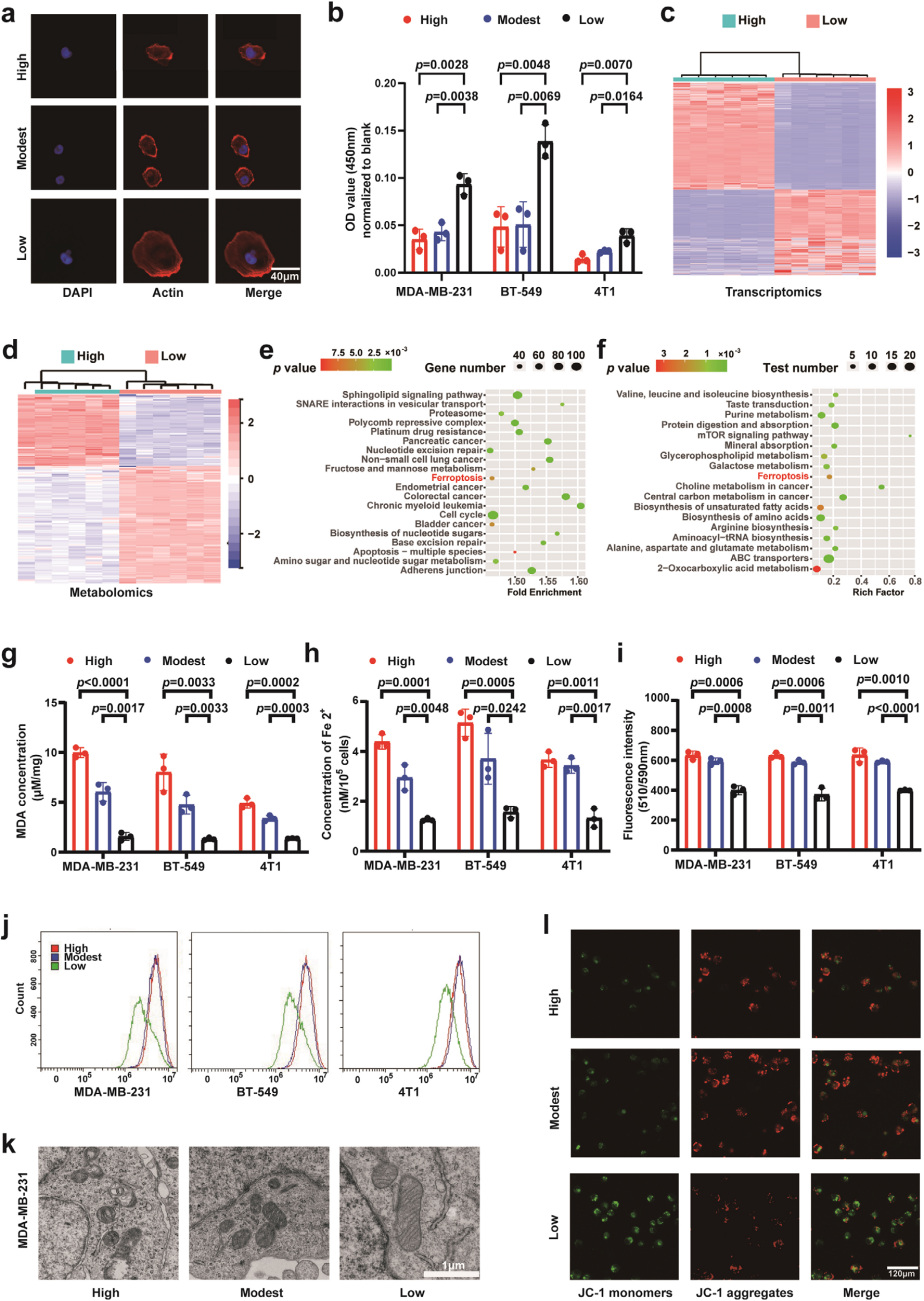
High‐density type I collagen induces ferroptosis of TNBC cells. (a) The actin fiber morphology of MDA‐MB‐231 cells cultured on type I collagen at different densities. Blue indicated DAPI, and Red indicated actin fiber. (b) CCK‐8 experiments in different TNBC cells (MDA‐MB‐231, BT‐549, and 4T1) cultured on type I collagen at different densities. (c) The heatmap of transcriptomics study in MDA‐MB‐231 cells cultured on type I collagen at different densities. (d) The heatmap of metabolomics study in MDA‐MB‐231 cells cultured on type I collagen at different densities. (e) KEGG enrichment analysis of DEGs from MDA‐MB‐231 transcriptomics study. (f) KEGG enrichment analysis of DEMs from MDA‐MB‐231 metabolomics study. (g) Lipid formation of TNBC cells cultured on type I collagen at high/modest/low density as measured by MDA assay. (h) The Fe^2+^ level of TNBC cells cultured on type I collagen at high high/modest/low density was measured. (i,j) The lipid ROS level was assessed by flow cytometry using C11‐BODIPY. (k) The mitochondrial morphology of MDA‐MB‐231 cells cultured on type I collagen at high high/modest/low was photographed by TEM. (l) The mitochondrial membrane potential of MDA‐MB‐231 cells cultured on type I collagen at high high/modest/low density was measured by using a JC‐1 probe. Statistical significance was determined by two‐tailed unpaired t‐test (b,g–i). Unless otherwise specified, *n* = 3. All studies were performed with three biological replicates. Values are shown as the means ± SDs (b,g–i).

TNBC cells were cultured in the above‐mentioned in vitro model, and cells (MDA‐MB‐231, BT‐549, and 4T1) displayed divergent morphologies in terms of actin microfilament spreading and formation under different collagen densities (Figure 2a and Figure S2), suggesting mechanical engagement. Cells cultured on high‐density collagen had significantly lower viability than cells cultured on low‐density collagen (Figure 2b). Apoptosis, necrosis, proliferation, and colony formation assays were further conducted, and none of these parameters were different between high‐ and low‐density collagen (Figures S3–S5).

Transcriptomics and metabolomics of MDA‐MB‐231 cells cultured on high‐ or low‐density collagen displayed numerous differential genes (Figure 2c, Table S2) and metabolites (Figure 2d, Table S3). The Kyoto Encyclopedia of Genes and Genomes (KEGG) enrichment analysis of differentially expressed genes (DEGs) revealed the top 20 enriched signaling pathways, such as cell cycle, sphingolipid signaling pathway, ferroptosis, and nucleotide sugars biosynthesis, among others (Figure 2e). KEGG enrichment analysis of differentially expressed metabolites (DEMs) identified 18 enriched signaling pathways, including the mTOR signaling pathway, alanine, aspartate, and glutamate (Glu) metabolism, and ferroptosis, among others (Figure 2f). Pathways showing concordant alterations at both the transcriptional and metabolic levels are more likely to reflect genuine functional reprogramming. Therefore, we intersected the pathways significantly enriched in each omics layer. The result suggested that ferroptosis was the only co‐enriched signaling pathway. Thus, we speculated that high‐density type I collagen might affect the progression of TNBC through modulating ferroptosis. Then, TNBC cells were seeded into our in vitro models to detect ferroptosis‐related indicators. The results revealed that TNBC cells cultured on high‐density collagen displayed high levels of ferroptotic events, such as malondialdehyde (MDA) increase (Figure 2g), ferrous ion (Fe^2+^) accumulation (Figure 2h), lipid reactive oxygen species (ROS) production (Figure 2i,j), mitochondrial cristae swelling and ridge fracture (Figure 2k and Figure S6a), and mitochondrial membrane potential increase (Figure 2l and Figure S6b), compared with those on low‐density collagen.

### 2.3 High‐Density Type I Collagen Modulates Ferroptosis and Gln Accumulation in TNBC Cells

**FIGURE 3:**
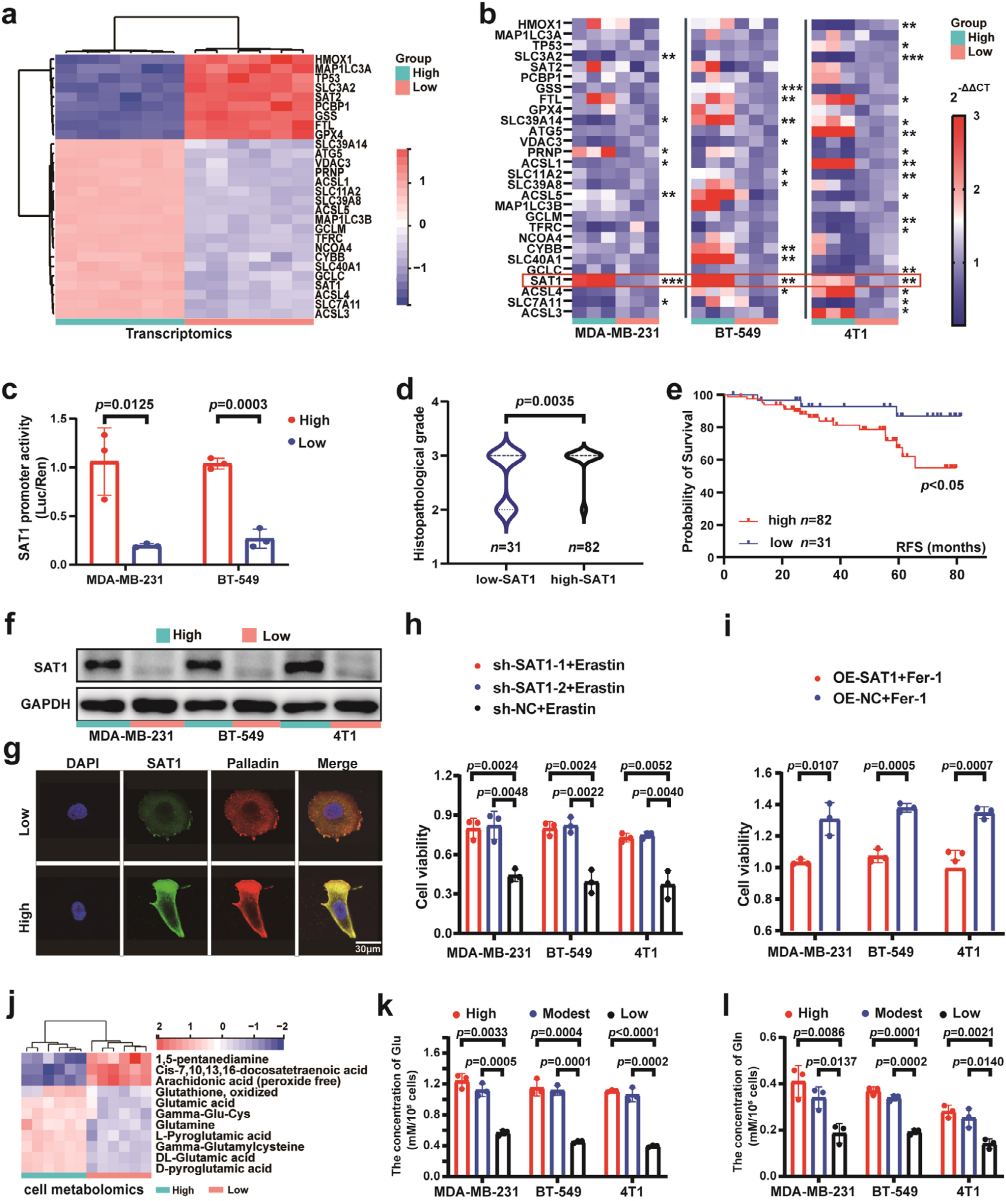
High‐density type I collagen accelerates ferroptosis and Gln accumulation in TNBC cells, probably via SAT1. (a) The heatmap of DFGs in the transcriptomics study of MDA‐MB‐231 cultured on type I collagen at different densities. (b) The heatmap of RT‐qPCR results of DFGs in TNBC cells (MDA‐MB‐231, BT‐549, and 4T1) cultured on type I collagen at different densities. (c) Differential analyses of SAT1 promoter activity in TNBC cells cultured on type I collagen at different densities. (d) Differential analyses of pathological grade in high‐ and low‐protein expression of SAT1 in TNBC. Violin plots showing data distribution. (e) The survival analysis of high‐ and low‐protein expression of SAT1 in TNBC. (f) Immunoblotting analysis of SAT1 in TNBC cells cultured on type I collagen at different densities. (g) IF analysis of SAT1 (in green) and palladin (in red) in MDA‐MB‐231 cultured on type I collagen at different densities. (h) Cells treated with erastin (5 µM) for 24 h, the cell viability was assessed by CCK‐8 assay. (i) Cells treated with Fer‐1 (1 µM) for 24 h, the cell viability was assessed by CCK‐8 assay. (j) The heatmap of DFMs in the metabolomics study of MDA‐MB‐231 cultured on type I collagen at different densities. (k) The level of Glu in TNBC cells cultured on type I collagen at different densities. (l) The level of Gln in TNBC cells cultured on type I collagen at different densities. Statistical significance was determined by two‐tailed unpaired t‐test (b,c,h,i,k,l), Kruskal‐Wallis H test (d), the two‐sided log‐rank (Mantel–Cox) test (e). Unless otherwise specified, *n* = 3. All studies were performed with three biological replicates. Values are shown as the means ± SDs (c,h,i,k,l).

Combining in vitro experiments and clinical data revealed that although high‐density type I collagen promoted TNBC ferroptosis, patients with high‐density type I collagen did not exhibit better outcomes. Studies have indicated that although ferroptosis regulates cell death, its effects on tumor promotion and suppression depend on altered damage‐related molecular profile and the immune response induced by ferroptosis in the TME [35, 36]. To explore the specific role of ferroptosis in high‐density collagen‐induced TNBC progression, differential ferroptosis‐related genes (DFGs) detected by transcriptomics studies are depicted in Figure 3a and Table S4. Real‐time quantitative Polymerase Chain Reaction (RT‐qPCR) was applied to confirm the expression of DFGs in TNBC cells cultured on different densities of type I collagen. The results indicated that the expression levels of spermidine/spermine N1‐acetyltransferase 1 (SAT1) were simultaneously increased in the three TNBC cell lines cultured on high‐density collagen compared with those on low‐density collagen (Figure 3b). SAT1 is the rate‐limiting enzyme regulating polyamine catabolism and participates in Gln metabolism and lipid peroxidation, which causes ferroptosis under ROS‐induced stress [37, 38]. To dissect how high‐density collagen modulates SAT1 transcription, we inserted the human SAT1 promoter into the pGL3‐Basic plasmid and performed dual luciferase reporter assays. The result showed that luciferase activity increased when TNBC cells were cultured on high‐density collagen gels versus low‐density collagen gels (Figure 3c), indicating that high‐density type I collagen stimulates the SAT1 promoter to enhance transcription.

SAT1 protein expression was evaluated by IHC in a tissue microarray (TMA) containing 245 breast cancer tissue samples. The chi‐squared test indicated that SAT1 protein expression was not associated with clinicopathological characteristics (age, T stage, pathological grade, lymph node metastasis, and Ki67 expression) in unstratified breast cancer (Table S5). However, SAT1 expression was closely related to pathological grade in TNBC (chi‐squared test *χ^2^* = 6.188, *p* = 0.0129; Table 4). Higher SAT1 expression levels were associated with higher pathological grades and lower RFS (Figure 3d,e), consistent with the roles of collagen density in TNBC tissues. Immunoblotting and immunofluorescence (IF) analyses revealed that TNBC cells cultured on high‐density collagen exhibited higher levels of SAT1 protein than those on low‐density conditions (Figure 3f,g and Figure S7a,b). To further investigate the roles of SAT1 in high‐density collagen‐induced ferroptosis of TNBC, SAT1‐silenced/overexpressed cell lines and the corresponding negative control (NC) were established, named sh‐SAT1‐1, sh‐SAT1‐2, sh‐NC, OE‐SAT1, and OE‐NC (Figure S7c–h). First, SAT1‐silenced or overexpressed cells were cultured in erastin‐ or Fer‐1‐containing media [5 µM erastin (ferroptosis inducer) and 1 µM Fer‐1 (ferroptosis inhibitor)] for 24 h, respectively, and cell viability was calculated using Cell Counting Kit‐8 (CCK‐8). The results indicated that SAT1‐silenced TNBC cells displayed decreased sensitivity to erastin (Figure 3h), whereas SAT1‐overexpressed cells exhibited lower sensitivity to Fer‐1 (Figure 3i), compared with the control.

**TABLE 4:** The associations between the expression of SAT1 and clinicopathologic features of TNBC patients.

		Total	The expression of SAT1			
	Factors	*n* = 102	Low *n* = 29	High *n* = 73	*χ2*	*p*
Age	<50	42	16	26	0.1311	ns
	≧50	60	25	35		
T stage	T1	39	12	27	3.865	ns
	T2	58	28	30		
	T3	5	1	4		
Pathological grade	II	19	12	7	6.188	0.0129
	III	72	23	49		
	Unknown	11	5	6		
Lymph node metastasis	0	62	24	38	0.1453	ns
	≧1	40	17	23		
Ki67	<30	9	3	6	0.216	ns
	≧30	92	38	54		
	Unknown	1	0	1		

Moreover, Figure 3j and Table S6 depict differential ferroptosis‐related metabolites (DFMs) of MDA‐MB‐231 cells identified by metabolomics. DFMs were all involved in the metabolism of Glu and Gln. Further quantification of Gln and Glu verified that TNBC cells on high‐density collagen had higher levels of both Glu and Gln than the controls (Figure 3k,l). Gln is recognized as indispensable for the proliferation of tumor cells in culture and plays “an intermediate” role in the tricarboxylic acid (TCA) cycle [39, 40]. The in vitro experiment in this study indicated that SAT1, a modulator of Gln metabolism, was up‐regulated in TNBC cells on high‐density collagen compared with those on low‐density collagen. Considering the contradictory roles of high‐density collagen in clinical samples and in vitro models, the mechanism may be mediated by the double‐edged sword effect of ferroptosis, regulated by SAT1‐induced Gln accumulation.

### 2.4 High‐Density Type I Collagen Promotes Gln Accumulation‐Related Ferroptosis Through SAT1/Argininosuccinate Synthase (ASS1)

**FIGURE 4:**
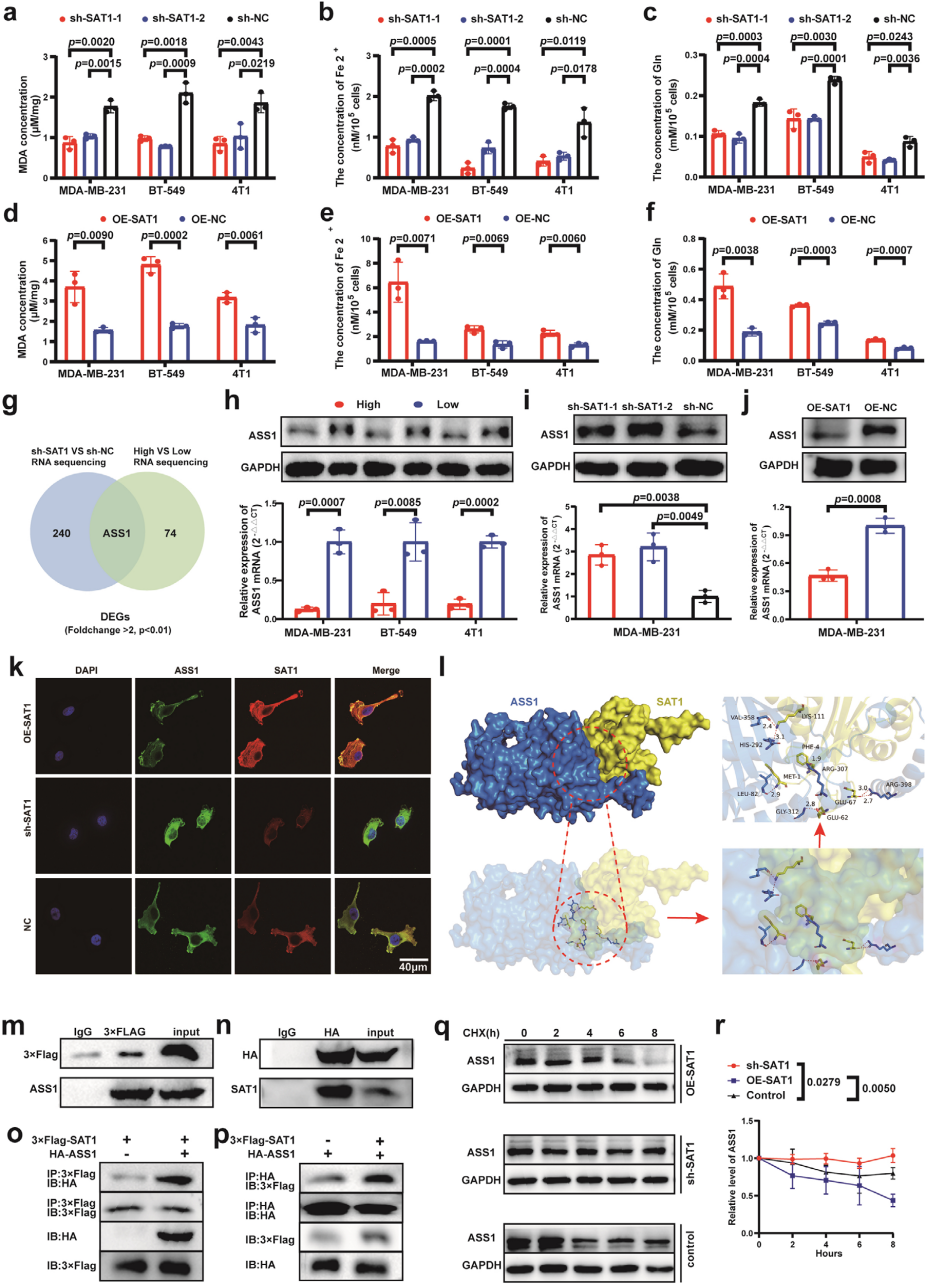
SAT1 induces TNBC ferroptosis and Gln accumulation through down‐regulating ASS1. Lipid formation (a,d), Fe^2+^ (b,e), and Gln level (c,f) were measured in indicated TNBC cells. (g) The Venn diagram of the RNA‐seq analysis of SAT1 deficiency and transcriptomics analysis of MDA‐MB‐231 in the in vitro collagen density model. (h) Immunoblotting analysis of ASS1 in TNBC cells cultured on type I collagen at different densities. (i,j) Immunoblotting analysis of ASS1 in indicated TNBC cells. (k) IF analysis of SAT1 and ASS1 in indicated cells. (l) The structural interaction of SAT1 and ASS1. (m) The 3× Flag‐SAT1 plasmid was transfected into HEK293T cells, and anti‐3× Flag IgG antibody and normal IgG were applied to conducted Co‐IP, respectively. The expression levels of ASS1 and 3× Flag‐SAT1 in whole cell lysis, immune‐precipitates produced by 3× Flag antibody, and by normal IgG were examined by immunoblotting. (n) The HA‐ASS1 plasmid was transfected into HEK293T cells, and anti‐HA IgG antibody and normal IgG were applied to conducted Co‐IP, respectively. The expression levels of SAT1 and HA‐ASS1 in whole cell lysis, immune‐precipitates produced by HA antibody, and by normal IgG were examined by immunoblotting. (o) The 3× Flag‐SAT1 and HA‐ASS1 plasmid were transfected into HEK293T cells as indicated, and anti‐3× Flag IgG antibody was applied to conduct Co‐IP. The expression levels of HA and 3× Flag were examined by immunoblotting. (p) The 3× Flag‐SAT1 and HA‐ASS1 plasmid were transfected into HEK293T cells as indicated, and anti‐HA IgG antibody was applied to conduct Co‐IP. The expression levels of HA and 3× Flag were examined by immunoblotting. (q) SAT1‐deficient, SAT1‐overexpressed and control MDA‐MB‐231 cells were treated with CHX for 8 h and collected every 2 h to examine the ASS1 levels by western blotting. GAPDH was used as a loading control. (r) Line chart presented the change of ASS1 protein level over time under CHX treatment. Statistical significance was determined by two‐tailed unpaired t‐test (a–f). Unless otherwise specified, *n* = 3. All studies were performed with three biological replicates. Values are shown as the means ± SDs (a–f,r).

To explore the association between SAT1 and Gln, SAT1‐silenced TNBC cells were cultured in a high‐density collagen model for 48 h. The results revealed that SAT1‐silenced cells exhibited less ferroptosis, as indicated by lower MDA and Fe^2+^ levels, than the NC (Figure S8a). Gln accumulation was synchronously decreased in SAT1‐silenced cells (Figure S8a). Additionally, silencing SAT1 in TNBC cells decreased MDA, Fe^2+^, and Gln levels (Figure 4a–c). Conversely, SAT1 overexpression in TNBC cells increased the levels of MDA and Fe^2+^, along with Gln levels (Figure 4d–f). Then, SAT1 was overexpressed in SAT1‐silenced cells to determine whether it could rescue ferroptosis inhibition caused by SAT1 silencing. Using a shRNA targeting the 3′ untranslated region (3′ UTR) of SAT1, SAT1‐silenced TNBC cell lines (sh‐SAT1‐3) were constructed. The engineered cDNA of SAT1 was reintroduced into sh‐SAT1‐3 cells to restore SAT1 expression levels (sh‐SAT1‐3 + OE). Western blotting validated the successful construction of the cell lines (sh‐SAT1‐3, sh‐SAT1‐3 + OE, sh‐NC) (Figure S8b–g). The results revealed that restoring SAT1 in sh‐SAT1‐3 cell lines rescued ferroptosis inhibition, accompanied by Gln accumulation (Figure S8h). This indicated that the mechanism potentially depended on SAT1‐regulated Gln accumulation‐related ferroptosis.

RNA‐sequencing (RNA‐seq) was conducted to further investigate the mechanisms by which the SAT1 regulates Gln accumulation. Venn diagrams were generated using the following data: 1) genes that were up‐ or down‐regulated by SAT1 knockdown in MDA‐MB‐231 in a RNA‐seq analysis, and 2) Gln metabolism‐related DEGs in the transcriptome of the in vitro collagen model of MDA‐MB‐231. Finally, ASS1 appeared to participate in SAT1‐regulated Gln metabolism (Figure 4g, Tables S7 and S8). ASS1, a sensor for the Gln deprivation response, promoted the reductive carboxylation of cytosolic Gln and compromised the oxidative TCA cycle from Gln anaplerosis, reducing mitochondrial‐derived lipid ROS [39]. The ASS1 expression levels in our in vitro collagen model were detected using Western blotting. The results indicated that protein expression levels were markedly decreased in TNBC cells cultured on high‐density collagen compared to those on low‐density (Figure 4h). Moreover, the protein expression levels of ASS1 were higher in SAT1‐silenced cells than those in NC (Figure 4i). SAT1‐overexpressed cells exhibited lower ASS1 levels than the NC (Figure 4j). IF staining of SAT1 and ASS1 proteins was also conducted on SAT1‐silenced, overexpressed, and NC cells to examine the reciprocal expression relationship between SAT1 and ASS1. Compared with the NC, the fluorescence intensity of ASS1 protein was obviously elevated in SAT1‐silenced cells, suggesting a concomitant increase of ASS1 expression after SAT1 silencing (Figure 4k). In contrast, the fluorescence intensity of ASS1 protein was decreased in SAT1‐overexpressing cells, indicating a concomitant decrease of ASS1 expression after SAT1 overexpression (Figure 4k).

To demonstrate SAT1‐ASS1 direct physical interaction, we performed in silico protein–protein docking using GRAMM. The analysis revealed a high‐confidence interface between SAT1 and ASS1, with a binding energy of −14.9 kcal mol^−^^1^, indicating a stable complex (Table S9). The surface model illustrated that the two proteins dock via complementary interfaces, where an extensive hydrogen‐bond network stabilizes the complex and markedly strengthens the binding affinity (Figure 4l). Subsequent mapping of key amino acid residues reveals that hydrogen bonds are formed at multiple contact points, thereby conferring additional structural stability to the SAT1‐ASS1 interaction. To confirm the physical interactions between SAT1 and ASS1, a Co‐IP analysis of SAT1 and ASS1 was performed. As demonstrated in Figure 4m, ASS1 was precipitated in 3× Flag‐SAT1‐transfected HEK293T cells by using an anti‐3 × Flag. Similarly, SAT1 was precipitated in HA‐ASS1‐transfected HEK293T cells by using an IgG antibody against HA (Figure 4n). Furthermore, HEK293T cells were co‐transfected with 3× Flag‐SAT1 and HA‐ASS1, and the results revealed that HA was precipitated in 3× Flag‐SAT1‐transfected HEK293T cells using an IgG antibody against 3× Flag (Figure 4o). Additionally, 3× Flag was precipitated in HA‐ASS1‐transfected HEK293T cells using an IgG antibody against HA (Figure 4p). These data suggested that the expression of ASS1 was regulated by SAT1. To further verify the regulatory effect of SAT1 on the stability of the ASS1 protein, we performed a time‐course experiment to track ASS1 protein degradation in cycloheximide (CHX)‐treated cells [41]. As shown in Figure 4q, the level of ASS1 remained unchanged for 8 h following CHX treatment in SAT1‐silenced MDA‐MB‐231 cells. However, in control MDA‐MB‐231 cells, the level of ASS1 dramatically declined within 8 h following CHX treatment. In SAT1‐overexpressed MDA‐MB‐231 cells, the level of ASS1 showed a gradual decline over 8 h in the presence of CHX. Quantitative time‐course data were summarized in Figure 4r. These findings indicated that SAT1 shortened ASS1 half‐life by accelerating its degradation.

Additionally, ASS1‐silenced (sh‐ASS1‐1 and sh‐ASS1‐2) and ASS1‐overexpressed (OE‐ASS1) cell lines were generated, and ASS1 protein expression was evaluated (Figure S9a–f). ASS1‐overexpressed cells were seeded in our in vitro high‐density collagen model, revealing that ASS1 overexpression rescued high‐density collagen‐induced ferroptosis, accompanied by decreased Gln levels (Figure S9g). ASS1 knockdown or overexpression modulated ferroptosis and Gln accumulation (Figure S9h,i). For the rescue experiment, ASS1 was overexpressed in SAT1‐overexpressing cells, and ASS1 was knocked down in SAT1‐deficient cells, to detect ferroptotic markers. The results revealed that overexpressing ASS1 rescued SAT1‐induced ferroptosis and Gln accumulation, whereas silencing ASS1 reversed SAT1 knockdown‐induced ferroptosis resistance and decreased Gln levels (Figure S9j–o). Therefore, ferroptosis regulation driven by high‐density type I collagen was likely dependent on SAT1‐ASS1‐associated Gln accumulation.

### 2.5 High‐Density Type I Collagen Advances Gln Accumulation‐Related Ferroptosis and Interferon‐Gamma Positive (IFN‐γ^+^) CD8^+^ T Cell Exhaustion In Vivo

**FIGURE 5:**
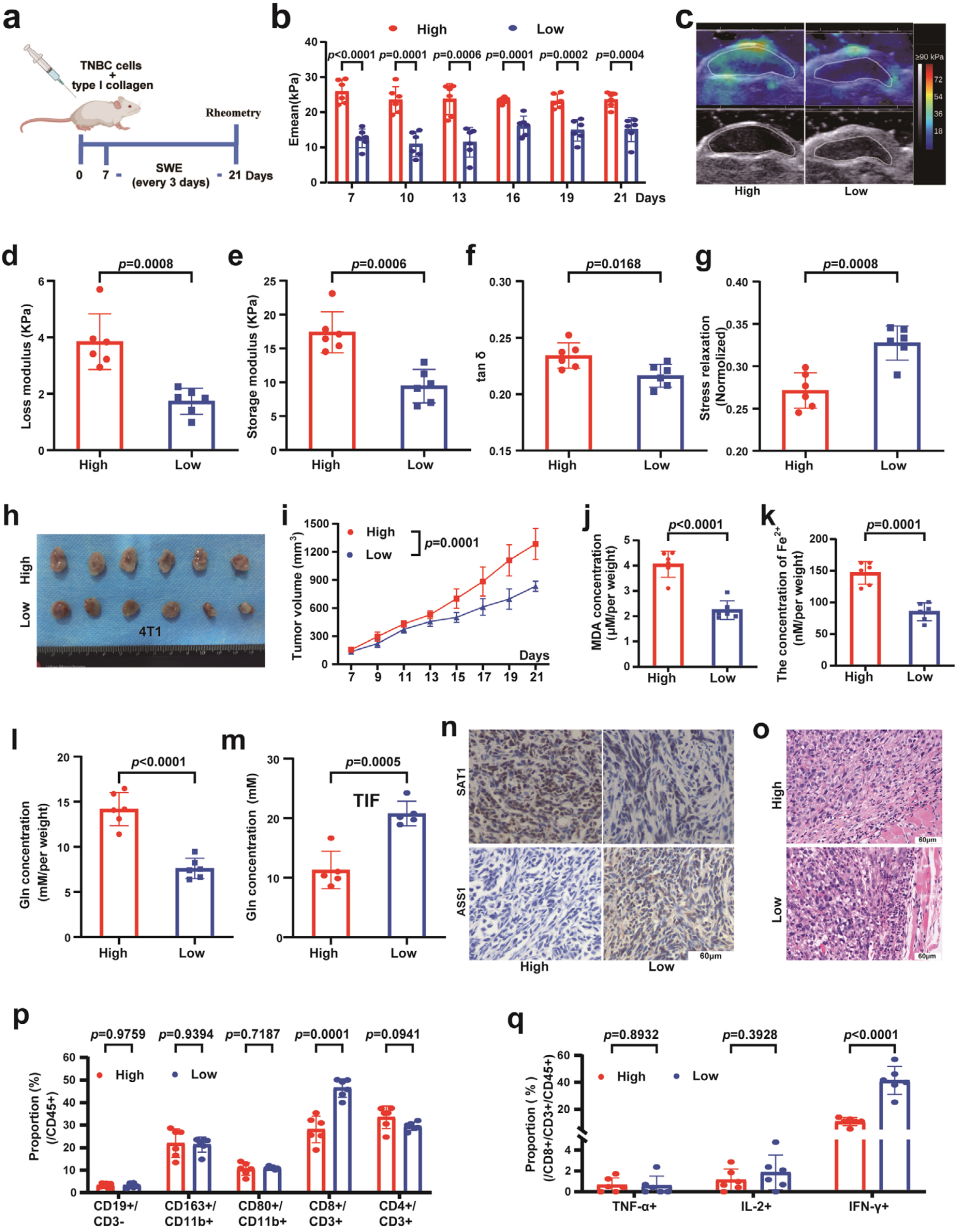
High‐density type I collagen promotes Gln accumulation–related ferroptosis and IFN‐γ^+^ CD8^+^ T cells exhaustion in vivo. (a) The establishment and verification pattern of the in vivo collagen density model of 4T1. (b) The SWE Emean values of tumors during the establishment process of the in vivo collagen density model. *n* = 6. (c) The image of SWE Emean value of tumors in the in vivo collagen density model at day 21. The loss modulus (d), storage modulus (e), tanδ (f), and stress relaxation (g) of tumors in the in vivo collagen density model at day 21. *n* = 6. (h) The image of tumors in the in vivo collagen density model. *n* = 6. (i) The growth curve of tumors of the in vivo collagen density model. *n* = 6. Lipid formation (j), Fe^2+^ (k), Gln (l), and TIF Gln (m) levels were examined in tumors of the in vivo collagen density model. *n* = 6. (n) IHC images of both SAT1 and ASS1 in the in vivo collagen density model. (o) The proportion of TILs in the TME of the in vivo collagen density model. (p) The proportion of immune cells of the in vivo collagen density model. *n* = 6. (q) The proportion of intracellular cytokines in CD8^+^ T cells of the in vivo collagen density model. *n* = 6. Statistical significance was determined by two‐tailed unpaired t‐test (b,d–g,j–m,p,q) and two‐way analysis of variance (ANOVA) (i). Values are shown as the means ± SDs (b,d–g,i–m,p,q).

Since the effects of ferroptosis on tumors depend on the specific changes of molecular patterns and immune responses caused by ferroptosis within the TME, taken together the results of in vitro experiments and clinical samples, we hypothesized that in the process of high‐density collagen‐induced ferroptosis, TNBC cells take up a large quantity of Gln through the SAT1‐ASS1 signaling pathway, which may lead to the shortage of Gln for immune cells in the TME, thus contributing to poor patient outcomes. To verify our theory, an in vivo model was established by transplanting a mixture of TNBC cells and type I collagen solution (2.5 mg mL^−1^ or 50 µg mL^−1^) (Figure 5a). The tumor mechanical features were monitored using SWE (every three days) and rheometry (at sacrifice). As illustrated in Figure 5b–g and Figure S10a–f, tumors derived from the high‐concentration collagen mixture exhibited higher Emean values, G′, G″, and tan δ, and faster stress relaxation, than those derived from a low‐concentration collagen mixture, suggesting that tumors developed from the high‐concentration collagen had more pronounced biomechanical properties. The progression of tumors with high‐density collagen was significantly faster than that of tumors with low‐density collagen (Figure 5h,i and Figure S10g,h). The ferroptosis‐related indicators revealed that tumors with high‐density collagen had higher levels of MDA and Fe^2+^ (Figure 5j,k and Figure S10i,j). The concentration of Gln was obviously elevated in tumors with high‐density collagen, while it was decreased in tumor interstitial fluid (TIF) with high‐density collagen, compared with tumors with low‐density collagen (Figure 5l,m and Figure S10k,l). IHC staining demonstrated that high‐density collagen induced the up‐regulation of SAT1 and the down‐regulation of ASS1 (Figure 5n and Figure S10m). Immune cell infiltration in the TME was evaluated to explore the associations between Gln‐induced ferroptosis and immunology response. There was no difference in immune cell infiltration between high‐ and low‐density collagen (Figure 5o). However, tumors with high‐density collagen had a lower proportion of CD8^+^ T cells than those with low‐density collagen (Figure 5p and Figure S10n–p). In contrast, B cells, M1/M2 macrophages, and CD4^+^ T cells exhibited no difference (Figure 5p and Figure S10p). Further tests of key cytokines revealed that CD8^+^ T cells in tumors with high‐density collagen expressed lower levels of IFN‐γ compared with those with low‐density collagen, while interleukin‐2 (IL‐2) and tumor necrosis factor α (TNF‐α) exhibited no difference (Figure 5q and Figure S10q–s). TNBC cells cultured on high‐density collagen were also co‐cultured with CD8^+^ T cells, and the levels of Gln and IFN‐γ in the media were assessed (Figure S11a). The results indicated that TNBC cells cultured on high‐density collagen deprived CD8^+^ T cells of Gln and inhibited the secretion of IFN‐γ by CD8^+^ T cells (Figure S11b,c). In addition, after co‐culture, the viability of TNBC cell cultured on high‐density collagen was markedly higher than those on low‐density collagen (Figure S11d), a trend that was completely reversed when TNBC cells were cultured independently in our in vitro collagen density models (Figure 2b). To delineate the role of Gln deprivation in IFN‐γ^+^ CD8^+^ T cell exhaustion, CD8^+^ T cells were cultured in Gln‐free, Gln‐replete, or Gln‐excess medium, followed by assessment of IFN‐γ secretion. IFN‐γ secretion by CD8^+^ T cells was markedly reduced under Gln‐free conditions and elevated under Gln‐excess conditions relative to Gln‐replete controls (Figure S11e). Additionally, CD8^+^ T cells were co‐cultured with TNBC cells in which SAT1 was stably silenced or overexpressed (Figure S11f). As shown in Figure S11g–i, SAT1 knock‐down attenuated Gln uptake in TNBC cells, preserved extracellular Gln levels, and stimulated IFN‐γ secretion by CD8^+^ T cells. Conversely, SAT1 overexpression enhanced Gln uptake, depleted extracellular Gln, and inhibited IFN‐γ secretion by CD8^+^ T cells (Figure S11j–l).

**FIGURE 6:**
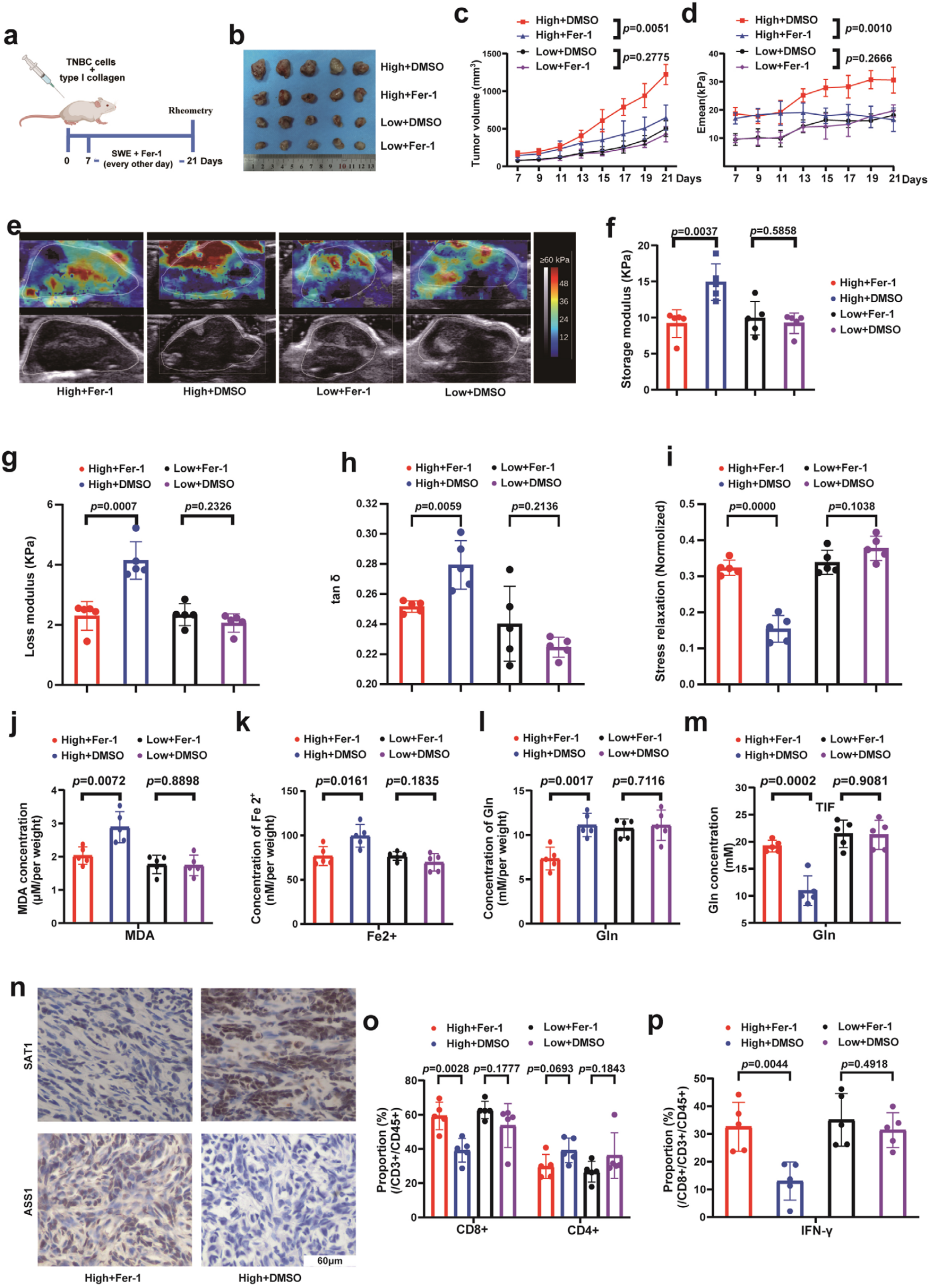
The treatment of Fer‐1 in the in vivo high‐density type I collagen model. (a) The pattern of dosage regimen in the in vivo collagen density model (b) The image of tumors treated with indicated drugs in the in vivo collagen density model at day 21. *n* = 5. (c) The growth curve of tumors treated with indicated drugs in the in vivo collagen density model. *n* = 5. (d) The curve of SWE Emean values of tumors treated with indicated drugs. *n* = 5. (e) The images of SWE in tumors treated with indicated drugs at day 21. The storage modulus (f), loss modulus (g), tan δ (h), and stress relaxation (i) of tumors in the in vivo collagen density model at day 21. *n* = 5. Lipid formation (j), Fe^2+^ (k), Gln (l), and TIF Gln (m) levels were examined in tumors treated with indicated drugs. *n* = 5. (n) IHC images of both SAT1 and ASS1 in tumors treated with indicated drugs. (o) The proportion of CD4^+^ and CD8^+^ T cells in tumors treated with indicated drugs. *n* = 5. (p) IFN‐γ expression in CD8^+^ T cells of tumors treated with indicated drugs. *n* = 5. Statistical significance was determined by two‐way analysis of variance (ANOVA) (c) and two‐tailed unpaired t‐test (d,f–m,o,p). Values are shown as the means ± SDs (c,d,f–m,o,p).

To explore the association between high‐density collagen‐induced ferroptosis and IFN‐γ^+^ CD8^+^ T cell exhaustion, CD8^+^ T cells were cultured for 24 h in either Fer‐1‐containing medium or standard medium. Cell viability was assessed using the CCK‐8 assay, and no significant difference was observed between the two groups. (Figure S12a). Then, Fer‐1 was injected every other day into the in vivo collagen model (Figure 6a). Tumors with high‐density collagen treated with Fer‐1 (tumor ^High/Fer‐1^) exhibited restrained tumor progression (Figure 6b,c), with lowered Emean values, G′, G′′ and tanδ, and slower stress relaxation, compared to those treated with control (tumor ^High/DMSO^)(Figure 6d–i), whereas this phenomenon was not observed in those with low‐density collagen (tumor ^Low/Fer‐1^ versus tumor ^Low/DMSO^) (Figure 6b–i). In addition, tumors ^High/Fer‐1^ displayed a decreased level of ferroptosis, indicated by MDA and Fe^2+^, a lowered level of Gln, and a higher level of Gln in TIF, compared with tumors ^High/DMSO^, while the phenomenon was also not observed in those of low‐density collagen (tumor ^Low/Fer‐1^ versus tumor ^Low/DMSO^) (Figure 6j–m). The protein expression levels of SAT1 and ASS1 in tumors were detected by IHC. SAT1 was significantly down‐regulated in tumor ^High/Fer‐1^, accompanied by ASS1 elevation, compared with tumor ^High/DMSO^ (Figure 6n). The proportion of CD8^+^ T cells was elevated in tumors ^High/Fer‐1^, but not CD4^+^, compared to those of tumors ^High/DMSO^. However, no difference was evident between tumors ^Low/Fer‐1^ and tumors ^Low/DMSO^ (Figure 6o and Figure S12b). Furthermore, IFN‐γ expression in CD8^+^ T cells was significantly elevated in tumors ^High/Fer‐1^, compared to tumors ^High/DMSO^, with no difference between tumors ^Low/Fer‐1^ and tumors ^Low/DMSO^ (Figure 6p and Figure S12c).

The co‐culture and in vivo drug intervention experiments revealed that high‐density type I collagen promoted IFN‐γ^+^ CD8^+^ T cell exhaustion by inducing Gln accumulation–related ferroptosis.

### 2.6 High‐Density Type I Collagen Promotes IFN‐γ^+^ CD8^+^ T Cell Exhaustion in the TME Through SAT1‐ASS1‐Induced Gln Accumulation in Tumor Cells In Vivo

**FIGURE 7:**
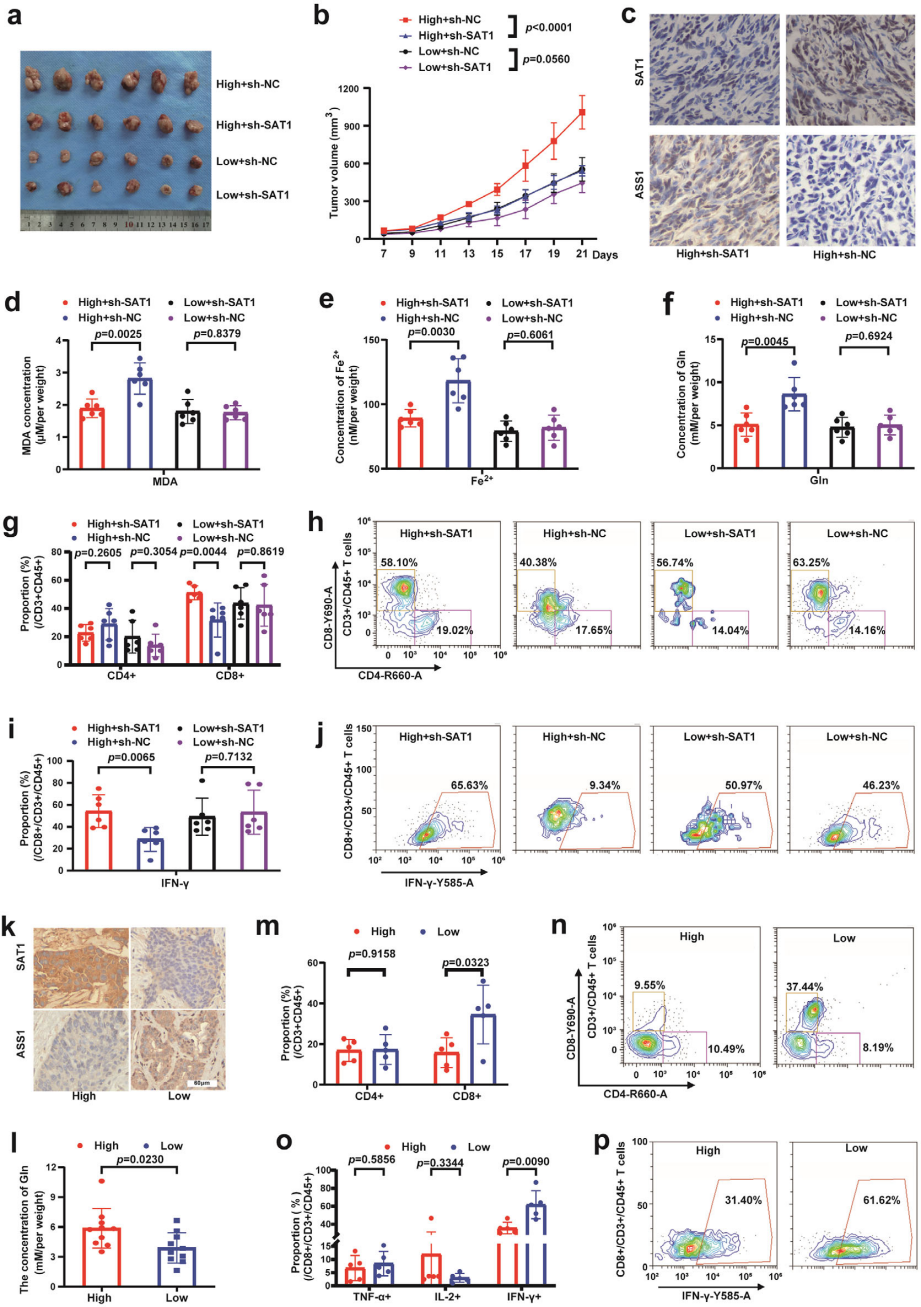
High‐density type I collagen promotes IFN‐γ^+^ CD8^+^ T cell exhaustion in vivo through SAT1–ASS1‐regulated Gln accumulation–related ferroptosis. (a) Image of tumors in the indicated in vivo collagen density model. *n* = 6. (b) The growth curve of tumors in the indicated in vivo collagen density model. *n* = 6. (c) IHC images of SAT1 and ASS1 in the indicated in vivo collagen density model. Lipid formation (d), Fe^2+^ (e), and Gln (f) levels were examined in tumors of the indicated in vivo collagen density model. *n* = 6. (g,h) The proportion of CD4^+^ and CD8^+^ T cells in the TME of the indicated in vivo collagen density model. *n* = 6. (i,j) IFN‐γ expression in CD8^+^ T cells of the indicated in vivo collagen density model. *n* = 6. (k) IHC images of SAT1 and ASS1 in lesions of patients with TNBC of high or low collagen expression. (l) The Gln level was examined in lesions of patients with TNBC of high or low collagen expression. *n* = 10. (m,n) The proportion of CD4^+^ and CD8^+^ T cells in lesions of patients with TNBC of high or low collagen expression. *n* = 5. (o,p) IFN‐γ expression in CD8^+^ T cells in lesions of patients with TNBC of high or low collagen expression. *n* = 5. Statistical significance was determined by two‐way analysis of variance (ANOVA) (b) and two‐tailed unpaired t‐test (d–g,i,l,m–o). Values are shown as the means ± SDs (b,d–g,i,m,l–o).

Silencing SAT1 in the high‐density in vivo collagen model (tumor ^High/sh‐SAT1^) inhibited tumor progression compared to tumor ^High/sh‐NC^, while silencing SAT1 in the low‐density in vivo collagen model (tumor ^Low/sh‐SAT1^) showed no difference in tumor growth compared with tumor ^Low/sh‐NC^ (Figure 7a,b). Furthermore, SAT1 deficiency up‐regulated ASS1 protein expression levels in tumor ^High/sh‐SAT1^ compared to those in tumor ^High/sh‐NC^ (Figure 7c). Corresponding levels of MDA and Fe^2+^ were decreased in tumor ^High/sh‐SAT1^ compared to tumor ^High/sh‐NC^, and were accompanied by decreased Gln levels (Figure 7d–f). Evaluation of CD4^+^ and CD8^+^ T cell infiltration indicated that tumor ^High/sh‐SAT1^ displayed higher CD8^+^ T cell infiltration, but not CD4^+^ T cell infiltration, than tumor ^High/sh‐NC^ (Figure 7g,h). Additionally, elevated levels of IFN‐γ in CD8^+^ T cells were observed in tumor ^High/sh‐SAT1^ (Figure 7i,j). These results suggested that high‐density type I collagen may trigger IFN‐γ^+^ CD8^+^ T cell exhaustion in the TME through SAT1‐ASS1‐induced Gln accumulation‐related ferroptosis of TNBC cells.

The SAT1, ASS1, and Gln levels, and the proportion of IFN‐γ^+^ CD8^+^ T cells were also analyzed in tumor tissues with different collagen expression characteristics in patients with TNBC. IHC analysis revealed that tumors with high collagen expression shared higher levels of SAT1 and lower levels of ASS1, accompanied by Gln accumulation, compared to those with low collagen expression (Figure 7k,l). Further evaluation of IFN‐γ^+^ CD8^+^ T cells indicated that tumors with high collagen expression demonstrated a lower accumulation of IFN‐γ^+^ CD8^+^ T cells than those with low collagen expression (Figure 7m–p).

### 2.7 Combination of Fer‐1 and Anti‐PD‐1 Therapy Reverses Type I Collagen‐Induced Tumor Progression of TNBC

**FIGURE 8:**
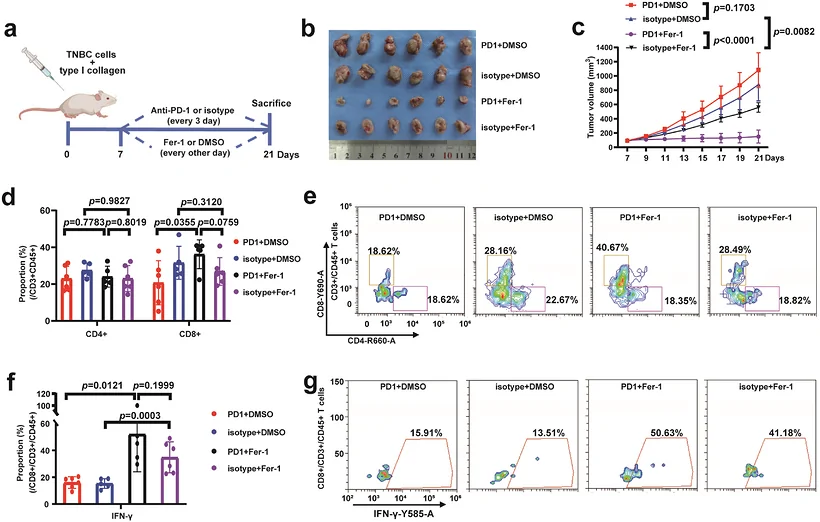
Combination of Fer‐1 and anti‐PD‐1 therapy reverse high‐density type I collagen‐induced tumor progression of TNBC. (a) The pattern of dosage regimen in the in vivo collagen density model of 4T1. (b) Image of tumors in the indicated in vivo collagen density model. *n* = 6. (c) The growth curve of tumors in the indicated in vivo collagen density model. *n* = 6. (d,e) The proportion of CD4^+^ and CD8^+^ T cells in the TME of the indicated in vivo collagen density model. *n* = 6. (f,g) IFN‐γ expression in CD8^+^ T cells of the indicated in vivo collagen density model. *n* = 6. Statistical significance was determined by two‐way analysis of variance (ANOVA) (c,j) and two‐tailed unpaired t‐test (d,f). Values are shown as the means ± SDs (c,d,f,j).

To test the effect of immunotherapy in high‐density type I collagen‐related immunosuppression, we first injected the mixture of 4T1 cells and 2.5 mg mL^−1^ type I collagen solution into BALB/c mice to conduct a high collagen density model. Seven days later, Fer‐1, anti‐PD‐1 antibody, isotype control, or DMSO were intraperitoneally injected into mice as illustrated in Figure 8a. The combination of Fer‐1 and anti‐PD‐1 antibody significantly suppressed tumor growth compared to Fer‐1 or anti‐PD‐1 therapy alone (Figure 8b,c). Flow cytometry analysis suggested that the proportion of CD8^+^ T cells was significantly elevated in the combination therapy group, compared to anti‐PD‐1 therapy alone, while the proportion of CD4^+^ T cells showed no significant difference (Figure 8d,e). Furthermore, IFN‐γ^+^ CD8^+^ T cells were observed at higher levels in the combination treatment group than the anti‐PD‐1 or Fer‐1 therapy groups (Figure 8f,g). Collectively, these preclinical models demonstrate that the combination of Fer‐1 and anti‐PD‐1 therapy reverses high‐density collagen‐associated tumor progression of TNBC.

## 3 Discussion

This study focused on the role of type I collagen density in the progression of TNBC using clinical samples and established in vitro and in vivo type I collagen density models. It was found that higher collagen density and associated mechanical properties potentially predicted higher pathological grades and poorer RFS in patients with TNBC. This is clinically important as increased collagen deposition can be a risk factor for forecasting more invasive characteristics of TNBC. Additionally, increased type I collagen density induced Gln accumulation‐related ferroptosis of TNBC cells via the SAT1‐ASS1 signaling pathway. The process deprived immune cells of Gln supply and induced IFN‐γ^+^ CD8^+^ T cell exhaustion in the TME (Graphical Abstract). This may explain the poor prognosis in patients with TNBC exhibiting elevated collagen density. Although the effect of collagen density on cancer progression has been studied, this study is the first, to our knowledge, to demonstrate how changes in type I collagen density affect TNBC progression and identify the complex role of collagen density‐induced ferroptosis‐related immune exhaustion in TNBC.

Over the last decade, ferroptosis, an iron‐dependent programmed cell death (PCD) induced by lipid peroxidation, has been discovered to participate in the tumorigenesis, development, and treatment response [42]. Conceptually, since ferroptosis is a metabolic form of PCD driven by oxidative stress, cancer cells may be prone to undergo ferroptosis under higher ROS load [43]. ROS‐induced ferroptosis led to more rapid tumor elimination both in vitro and in vivo [44]. For example, the elevated O‐GlcNAc transferase (OGT), a key sensor for ROS during ferroptosis, contributed to tumorigenesis and resistance to chemoradiotherapy in hepatocellular carcinoma (HCC) [45]. However, cancer cells may also utilize specific alterations of gene expression profiles to respond to these metabolic and oxidative stress. For example, activating transcription factor 4 (ATF4), one of stress effectors, induced solute carrier family 7a member 11 (SLC7A11) to block stress‐related ferroptosis, but suppressing hepatocarcinogenesis [46]. Liu et al. [47] also described the dual role of ferroptosis in enhancing radiation sensitivity while facilitating radiation‐induced injury in tumors. The mechanisms might depend on the specific alterations of gene expression profile and the immunology response induced by ferroptosis within the TME [35, 36]. For example, in melanoma, TYRO3 upregulation shields tumor cells from ferroptosis and thereby confers resistance to α‐PD‐1/PD‐L1 immune checkpoint blockade [48]. Targeting the Wnt/β‐catenin–MITF axis to promote ferroptosis in tumor cells markedly boosts the effectiveness of anti‐PD‐1 immunotherapy [49]. Our study revealed that high‐density type I collagen promoted ferroptosis of TNBC cells through SAT1‐ASS1‐dependent Gln accumulation, while patients with tumors of high collagen expression exhibited poorer survival. Further exploration revealed that the ferroptosis of TNBC cells deprived immune cells of Gln supply and induced IFN‐γ^+^ CD8^+^ T cell exhaustion in the TME. These observations underscore that the role of ferroptosis‐as either suppressor or promoter‐is dictated by the specific cell type within the lipid‐peroxidation network, the redox‐buffering capacity of adjacent stromal and immune cells, and the spatiotemporal context of cell death.

Gln is a key source of reduced nitrogen for biosynthetic reactions and a carbon source for replenishing the TCA cycle [50]. Recent studies have demonstrated that the role of Gln in cancer is more complicated than previously recognized, as the synthesis, uptake, and use of Gln are highly flexible and relies on cell types, oncogenic drivers, and the TME in cancers [51]. Traditionally, cancer cells are dependent on Gln for growth and proliferation; hence, blocking Gln metabolism may be effective in treating cancers [51]. For instance, Gln supports pancreatic ductal adenocarcinoma (PDAC) growth through a Kirsten rat sarcoma viral oncogene homologue (KRAS)‐regulated metabolic pathway [52], and Gln antagonist significantly decreased tumor growth in several in vivo PDAC models [53]. However, Fu et al. (2019) [54] revealed that high Gln consumption was associated with poor overall and cancer‐specific survival in patients with clear cell renal cell carcinoma (ccRCC) using The Cancer Genome Atlas (TCGA) data. The mechanism may be based on the fact that Gln consumption by ccRCC cells leads to the shortage of extracellular Gln, inducing the secretion of IL‐23 from tumor‐infiltrating macrophages (TIMs) through the up‐regulation of hypoxia‐inducible factor 1α. Accordingly, high‐Gln‐consumption tumors exhibit higher levels of immunosuppression [54].

As previously discussed, the role of ferroptosis in cancer depends on the specific alterations of gene expression profile and immune responses induced by ferroptosis in the TME. The seemingly contradictory results of our in vitro and in vivo experiments can be attributed to Gln accumulation in TNBC cells and local deprivation of extracellular Gln in the TME. Further in vitro and in vivo experiments verified our hypothesis that ferroptosis could induce immune exhaustion in the TME. In detail, high‐density type I collagen increased the uptake of Gln in TNBC cells by SAT1‐ASS1‐induced ferroptosis, leading to Gln deprivation of IFN‐γ^+^ CD8^+^ T cells in the TME.

Finally, several important limitations of this research need to be considered. First, it is essential to acknowledge that varying collagen density in vitro concurrently alters matrix stiffness, viscoelasticity, ligand density, and pore architecture. The mechanical properties of our in vitro collagen gels (<1 kPa) differ substantially from those of native breast tumor tissues (0.5–100 kPa). This discrepancy could potentially stem from the absence of additional in vivo ECM components (e.g., hyaluronic acid, elastin, proteoglycans) in reconstituted collagen gels, along with variations in testing parameters between soft gel and tissue rheology [55, 56]. Consequently, our in vitro and in vivo collagen mixture models do not fully recapitulate the mechanical microenvironment of breast tumors; rather, they serve as models to investigate how increased type I collagen density affects cancer cell behavior and immune interactions. Second, our mechanism exploration began with transcriptomics and metabolomics. Cross‐linking mass spectrometry, cleavage under targets and release using nuclease (CUT&RUN), and chromatin immunoprecipitation (ChIP‐seq) followed by transcriptomics and metabolomics might be applied to capture specific collagen density‐induced changes in protein interactomes. Third, considering the complex role of collagen density in the TME, many different signaling and metabolic pathways identified through transcriptomics and metabolomics studies may be worth additional investigation. Fourth, with the rapid progress of spatial transcriptomics and single‐cell RNA‐seq, these techniques should be applied to comprehensively depict changes in collagen density in the TME. Fifth, while the tunable model allows collagen density to be modulated, increasing collagen density unavoidably raises integrin‐binding ligand availability and alters pore architecture. Although we have acknowledged these concurrent changes throughout the manuscript, further rigorous studies are still needed to precisely clarify the independent role of individual mechanical parameters. Such follow‐up approaches include maintaining constant collagen ligand density by blending collagen with non‐adhesive polymer scaffolds, utilizing CRISPR‐modified collagen variants that retain intact mechanical properties while attenuating integrin‐binding capacity. Sixth, differences in strain magnitude, oscillation frequency, and testing fixtures applied during rheological measurement collectively contribute to the numerical disparity in G′ and G″ between in vitro hydrogels and in vivo tumor tissues. In future investigations, we intend to unify rheological testing parameters and optimize the experimental system to reduce such mechanical discrepancies. Seventh, although the combination of Fer‐1 and anti‐PD‐1 therapy reversed high‐density collagen‐promoted tumor progression of TNBC in our study, the potential interference of Fer‐1 with combination therapy needs more exploration. For example, previous studies indicated that Fer‐1 alone could alter oxidized phosphatidylcholine (OxPC) release, restore T‐cell proliferation and function in co‐culture, and inhibit tumor growth in vivo [57]. The combination of Fer‐1 and anti‐PD‐1 therapy might have unexpected effects in the progression of TNBC. We should carefully explore the specific role of Fer‐1 in TNBC to avoid interference with ROS‐ or ferroptosis‐based therapeutic mechanisms while preserving its cytoprotective role in normal tissues. Finally, our studies contained limited clinical samples; therefore, independent, multi‐centre cohorts should be prospectively enrolled to investigate the role of collagen density in the progression of TNBC.

## 4 Conclusion

The study revealed the role of collagen density and associated mechanical properties in predicting pathology grade and prognosis of TNBC. Increased type I collagen density promoted Gln accumulation‐related ferroptosis of TNBC cells via the SAT1‐ASS1 signaling pathway, thus depriving IFN‐γ^+^ CD8^+^ T cells of Gln supply and inducing immune exhaustion in the TME.

## 5 Experimental Section/Methods

### 5.1 Patient Samples

Between January and December 2019, 428 patients with breast cancer with no prior systemic neoadjuvant treatment were recruited for SWE examination at Fudan University Shanghai Cancer Center and the First Affiliated Hospital of Nanjing Medical University. The patient inclusion criteria were as follows: Age 18–90 years, pathological diagnosis of breast cancer, no other primary cancer, willingness to return for follow‐up, and lesions with a maximum diameter of less than or equal to 40 mm. A set of TMA samples from 245 breast cancer tissue samples diagnosed between August 2015 and December 2017 were collected at the first radical surgery with no prior systemic neoadjuvant treatment at Fudan University Shanghai Cancer Center. All patients were graded according to the Nottingham histologic scoring system [58]. RFS was defined as the interval between the date of surgery and the date of breast cancer recurrence or the last follow‐up (October 2024), whichever came first. The research protocols were approved by the Shanghai Cancer Center Institutional Review Board, and all patients provided written informed consent (Ethical number: 2107234‐18, 050432‐4‐2108*).

### 5.2 SWE

SWE was conducted using a supersonic aixplorer color doppler ultrasound diagnostic apparatus (Supersonic Imagine, Aixen Provence, France) with an SL 15‐4 MHz linear array transducer. The color range system was adjusted according to the specific position. Each tumor was measured three times, and the Emax, Emean, and shear wave data were recorded. All images were collected by two sonographers with 5–10 years of experience performing breast ultrasounds.

### 5.3 Rheometry Analysis of the Tumors

The rheometry analysis of the tumor tissues was based on the protocol by Weiguo Fan. et al.(2024) [10], which was slightly adjusted according to the characteristics of breast tumor tissues. Tissues were sliced 3 to 5 mm thick, placed on an 8 mm diameter parallel plate, and kept hydrated with DMEM during measurement. Both of G′ and G″ were recorded by ARES‐G2 rheometer (TA instruments, USA) using TA TRIOS software (version 5.5.0). During the measurement, the upper plate was gradually lowered to touch the tissue, and 0.01 N of initial force (0–300 Pa) was set to ensure appropriate contact of the tissue with the plate. Tests were taken with a dynamic time sweep test (0.3% strain constant, 1 rad s^−1^ oscillation frequency) for 100 s, then stress relaxation (1% initial strain, measurements taken for 100 s).

### 5.4 Cell Lines, Culture, and Reagents

TNBC cell lines (MDA‐MB‐231, BT‐549, and 4T1) and the human embryonic kidney cell line HEK293T were obtained from the American Type Culture Collection (Manassas, VA, USA). All MDA‐MB‐231, BT‐549, and 4T1 cell lines were authenticated using Short Tandem Repeat (STR) analysis by Shanghai Biowing Applied Biotechnology Co.Ltd. All MDA‐MB‐231, BT‐549, and HEK293T cell lines were cultured in Dulbecco's Modified Eagle Medium (DMEM, C11995500BT, Gibco, USA). The mouse TNBC cell line 4T1 was maintained in Roswell Park Memorial Institute (RPMI)‐1640 medium (C11875500BT, Gibco, USA). All media were supplemented with 10% fetal bovine serum (FBS, A5670701, Gibco, USA), 100 U mL^−1^ penicillin, and 100 µg mL^−1^ streptomycin (15070063, Gibco, USA) at 37°C in a humidified incubator with 5% CO_2_. The cell lines were checked for mycoplasma contamination weekly using the MycAway Plus‐Color One‐Step Mycoplasma Detection Kit (40612ES25, Yeasen, China). Cells were trypsinized using 0.25% trypsin‐ethylenediaminetetraacetic acid (trypsin‐EDTA, 25200072, Gibco, USA) and centrifuged at 800 × *g* for 3 min before resuspending in fresh media.

### 5.5 Construction and Characterization of In Vitro Collagen Density Model

The model was established by coating culture dishes with type I collagen (5005, Advanced BioMatrix, USA) at varying concentrations to generate different collagen densities. Low‐density substrates were generated by coating dishes with 50 µg mL^−1^ type I collagen solution and incubating at 37°C for 1 h to complete gelation. Modest‐ or high‐density substrates were generated by coating dishes with 1 or 2.5 mg mL^−1^ type I collagen solution and incubating at 37°C for 1 h. Additionally, the pH of different type I collagen solutions was adjusted to approximately seven, as indicated by the pH value tester using sodium hydroxide titration (0.1 N). Cells were seeded on top of the gels and incubated for 48 h after gently washing the dishes three times with phosphate buffer solution (PBS). When cells were collected from substrates, they were washed thrice with pre‐warmed Hank's Balanced Salt Solution (HBSS, 24020117, ThermoScientific, USA). Then, collagen gels were digested by adding pre‐warmed type I collagenase solution (1U in HBSS, 17100017, Gibco, USA) at 37°C for 20 min.

The viscoelastic properties of collagen hydrogels were measured using an ARES‐G2 rheometer (TA instruments, USA) with an 8 mm diameter plate, 1° cone angle, and 200 µm gap distance at 37°C. For hydrogel formation and gelation kinetics, collagen solution was poured onto an 8 mm diameter plate (chilled at 4°C), then heated from 4°C to 37°C for 5 s to promote hydrogel formation. The viscoelastic properties of the liquid‐to‐solid transition process and collagen hydrogels were recorded as G′ as an indicator of the elastic component, G″ as an indicator of the viscous component of the material using a 60‐min oscillation time step (1 Hz frequency, 1% strain amplitude, 37°C), then stress relaxation (1% initial strain, measurements taken for 100 s) [59]. To assess the mechanical stability of collagen hydrogels, precursor solutions were placed onto the 8 mm diameter plate (pre‐warmed at 37°C) for 60 min. Then, frequency steps were performed over a logarithmic scale from 0.1 to 10 Hz at 1% strain amplitude, and both G′ and G″ were recorded during the process.

### 5.6 Cell Actin Fluorescence Staining

Cells were fixed with 4% paraformaldehyde (G1101, Servicebio, China) for 10 min before being permeabilized in PBS with 0.5% Triton‐X100 (GC204003, Servicebio, China) for 5 min. Then, the cells were incubated in the dark with Actin‐Tracker Red‐555 (1:100, C2203S, Beyotime, China) at room temperature for 60 min. After incubation with 4′,6‐diamidino‐2‐phenylindole (DAPI), the samples were washed and covered with an antifade mounting medium (G1401, Servicebio, China) for microscopy. Images were acquired by using an FV3000 Laser scanning confocal microscope (Olympus, Japan).

### 5.7 CCK‐8 Assay

The cell viability was examined using the CCK‐8 (CK04, Dojindo, Japan) cell proliferation assay, according to the manufacturer's protocol. A total of 2000 cells per well were seeded and cultured in 100 µL of the media in 96‐well microplates pre‐covered with different collagen solutions. For the drug intervention experiment, cells were seeded and treated with drugs (erastin (5 µM, 329600, Sigma–Aldrich, USA), Fer‐1 (1 µM, SML0583, Sigma–Aldrich, USA), DMSO (0.02%, 20688, ThermoScientific, USA)), for 24 h. Absorbance at 450 nm was measured using a microplate reader (BioRad, USA) 2 h after adding 10 µL CCK‐8 reagent to each well.

### 5.8 Detection of Apoptosis and Necrosis by Annexin V‐FITC/PI Assay

Cell apoptosis and necrosis were assessed using an Annexin V‐FITC/PI Apoptosis and Necrosis Detection Kit (Cat. No. C1056, Beyotime Institute of Biotechnology, Shanghai, China). Cells were seeded in 6‐well plates at a density of 5 × 10^5^ cells per well and treated as indicated. After treatment, cells were harvested and washed twice with cold phosphate‐buffered saline (PBS). The cell pellet was resuspended in 1× Binding Buffer at a concentration of 1 × 10^6^ cells mL^−1^. Subsequently, 5 µL of Annexin V‐FITC and 5 µL of Propidium Iodide (PI) were added to 100 µL of the cell suspension and gently mixed. The cells were incubated in the dark at room temperature for 15 min. After incubation, 400 µL of 1× Binding Buffer was added to the mixture to bring the total volume to 500 µL. The stained cells were immediately analyzed by flow cytometry (BD FACSCalibur, BD Biosciences, San Jose, CA, USA) using the FL1 channel for Annexin V‐FITC and the FL2 channel for PI. Data were analyzed using FlowJo software (FlowJo LLC, Ashland, OR, USA).

### 5.9 EdU Assay

Cell proliferation was assessed using EdU Cell Proliferation Kit with AF488 (C0071S, Beyotime, China). Cells were plated in 6‐well plates at a density of 5 × 10^5^ cells per well. After 48 h of proliferation, 10 µM EdU was added to each well and incubated for 2 h at 37°C to label the proliferating cells. The cells were then fixed with 4% paraformaldehyde (G1101, Servicebio, China) and permeabilized with 0.3% Triton X‐100. Subsequently, Click Additive Solution was added to the cells and incubated for 30 min to develop the color. The cell nuclei were stained with a 1× Hoechst 33342 solution and incubated in the dark at room temperature for 10 min. The cells were run through a FACS flow cytometer (BD Biosciences, USA), and the resulting data were processed using CytExpert software.

### 5.10 Colony Formation Assay

Cells were seeded in 6‐well plates at 1000 cells per well and cultured for 7 to 14 days. The colonies were then fixed with 4% paraformaldehyde (G1101, Servicebio, China) for 10 min and stained with crystal violet staining solution (C0121, Beyotime, China) for 10 min. A colony was defined as a cluster containing more than 50 cells, and the colonies were counted under a microscope.

### 5.11 Transcriptomics

A total of 5 × 10^6^ cells were plated onto 100‐mm dishes covered with different collagen substrates and incubated for 48 h. Cells were lysed using TRIzol reagent (Invitrogen) and stored at −80°C. Transcriptomics was examined by Oebiotech Co. (Shanghai, China). The values of *p* ≤ 0.01 and |log_2_^FC^| ≥ 1 were considered to be statistically significant and identified as DEGs.

### 5.12 Metabolomics

A total of 1 × 10^7^ MDA‐MB‐231 cells were seeded into 100‐mm dishes covered with different collagen substrates and incubated for 48 h. Cells were collected after collagen substrates were digested with collagenase, washed with chilled PBS three times, and 1 mL of ice‐cold methanol‐acetonitrile‐water solution (methanol: acetonitrile: water = 2:2:1, v/v/v; CAS‐No:67‐56‐1, EMD Millipore Corporation; A955‐4, ThermoScientific; W6‐4, ThermoScientific) was added. Metabolites were rehydrated in 100 µL of 50% acetonitrile in liquid chromatography‐tandem mass spectrometry (LC‐MS/MS)‐grade water and centrifuged after vortexing to remove debris. The supernatant was separated using a hydrophilic interaction chromatography column of ultra‐high‐performance liquid chromatography (UHPLC, Agilent 1290 Infinity LC, USA). Liquid chromatography‐MS analysis of metabolites was performed using an AB Triple TOF 6600 Mass Spectrometer (Applied Biosystems SCIEX, USA). The XCMS software (version 3.10) was used for chromatogram review, nonlinear retention time alignment, matched filtration, and peak area integration. Collection of Algorithms ofMEtabolite pRofile Annotation (CAMERA) was used for annotation of isotopes and adducts. After normalization, the variable importance in the projection (VIP) value of each metabolites in the orthogonal partial least‐squares discriminant analysis (OPLS‐DA) model was calculated to show its contribution to the classification. The two‐tailed Student's t‐test was applied to assess the statistical differences between various metabolites. The values of *p* ≤ 0.01, VIP > 1, and |log_2_^FC^| ≥ 1 were considered to be statistically significant and identified as DEMs.

### 5.13 KEGG Enrichment Analysis

KEGG enrichment analyses of the DEGs and DEMs were performed with ClusterProfiler, MetaboAnalyst, enrichplot, and ggplot2 packages in R software (version 4.0.2) against the KEGG database (release 2024‐05‐01), using the entire set of annotated genes or metabolites as the background. The values of *p* ≤ 0.01 and FDR ≤ 0.025 were considered to be statistically significant.

### 5.14 MDA Assay

The relative MDA concentration in cell or tissue lysates was assessed using a lipid peroxidation assay kit (S0131, Beyotime, China) according to the manufacturer's instructions. Cells were seeded in a 100‐mm plate (5 × 10^6^ cells per plate), pre‐covered with different concentrations of collagen solutions, and cultured for 48 h. For the rescue experiment, cells were cultured for 48 h after silencing or overexpressing target genes. Cells or tissues were washed with ice‐cold PBS twice, lysed in ice‐cold RIPA buffer (P0013J, Beyotime, China), and centrifuged (12 000 × *g*, 15 min) to obtain the supernatant. BCA Protein Assay Kit (P0011, Beyotime, China) was used to determine the protein concentration of samples. MDA working solution (200 µL) was added to 100 µL supernatant or standard solution in different concentrations and incubated at 100°C for 15 min, then cooled to room temperature in a water bath and centrifuged at 1000 × *g* for 15 min. A total of 200 µL supernatant was pipetted into a 96‐well plate and analyzed using a microplate reader (BioRad, USA) to examine the absorbance at 532 nm.

### 5.15 Ferrous Iron Assay

The ferrous iron level was determined using the Ferrous Iron Assay Kit (E‐BC‐K881‐M for cell, E‐BC‐K773‐M for tissue; Elabscience Biotechnology, China), according to the manufacturer's instructions. The experimental design was the same as described in the MDA assay. Cells were collected, washed in ice‐cold PBS, and lysed in 200 µL buffer solution for 10 min on ice. Then, it was centrifuged at 13 000 × *g*, at 4°C for 10 min to remove insoluble material. A control solution (80 µL) was added to the control sample, and a chromogenic solution (80 µL) was added to the test or standard sample. This mixture was incubated at 37°C for 10 min. The absorbance of the mixture was measured at 593 nm using a microplate reader (BioRad, USA). For tissue ferrous iron detection, fresh tissue (0.1 g) was washed in ice‐cold PBS, homogenized in 600 µL extraction solution, then centrifuged at 12 000 × *g* at 4°C for 10 min to obtain supernatant. Chromogenic solution (150 µL) was added to 300 µL supernatant or standard solution and incubated at 37°C for 10 min. The mixture was centrifuged at 12 000 × *g* for 10 min to remove insoluble substances. The supernatant (300 µL) was pipetted into a 96‐well plate to examine the absorbance at 593 nm. The plate was protected from light during the entire incubation process.

### 5.16 Lipid ROS Assay

Lipid ROS levels were evaluated using BODIPY‐C11 (D3861, ThermoScientific, USA). The cells were seeded at a density of 2 × 10^5^/well in collagen‐pre‐covered 6‐well plates and cultured for 48 h. The cells were washed twice with PBS and re‐cultured with 2 mL fresh medium containing 5 µM BODIPY‐C11 for 20 min. Cells were collected and washed to remove excess BODIPY‐C11 and then resuspended in 1000 µL PBS. The cell suspension was filtered through a 0.4 µm cell strainer (431750, Corning, USA) and subjected to the flow cytometry (BD Biosciences). Oxidation of BODIPY‐C11 resulted in a shift of the fluorescence emission peak from 590 to 510 nm, which was proportional to lipid ROS generation.

### 5.17 Transmission Electron Microscopy (TEM)

Cell pellets (1 × 10^6^) were collected and fixed using 2.5% glutaraldehyde (P1126, Solarbio, China) for 24 h. Samples were post‐fixed in 1% osmic acid fixative for 2–3 h, rinsed with 0.1 M PBS three times for 5 min, dehydrated in gradual ethanol (30%–90%), embedded in epoxy resin (Eponate 12, Ted Pella, USA), and polymerized at 60°C for 72 h. The samples were sliced to 70 nm thickness using an ultra‐microtome (EMFC7, Leica, Germany) and stained with 3% uranium acetate and lead citrate. Finally, TEM (Tecnai Spiri, FEI, Japan) was used to observe and photograph.

### 5.18 Measurement of Mitochondrial Membrane Potential

The mitochondrial membrane potential was measured using the Mitochondrial membrane potential assay kit with JC‐1 (C2006, Beyotime, China). Cells were seeded at a density of 1 × 10^5^/well in a collagen‐pretreated confocal dish (801001, NEST, China) and cultured for 48 h. After coincubation, cells were washed twice with PBS and incubated with 1 mL of JC‐1 working solution at 37°C for 20 min, then rinsed with PBS to remove excess fluorochrome. An FV3000 Laser scanning confocal microscope (Olympus, Japan) was used for observation and photography.

### 5.19 RT‐qPCR

Total RNA was extracted from cells using TRIzol reagent (Invitrogen) according to the manufacturer's instructions. RNA was reverse transcribed into cDNA using PrimeScript RT Master Mix (6210A, TaKaRa, Japan) and subjected to RT‐qPCR by applying TB Green Premix Ex Taq II (RR420A, TaKaRa, Japan) on a Roche Light Cycler 480 II (Roche, Switzerland). The thermal cycling program was as follows: 95°C for 30 s; 40 cycles of 95°C for 5 s and 60°C for 34 s; 95°C for 5 s, and 60°C for 1 min; and then hold at 4°C. The expression of target genes was normalized to GAPDH or 18S rRNA and calculated using the 2^−ΔΔCt^ method. The primer sequences (5′–3′) are listed in Table S10.

### 5.20 Immunoblotting

Cells were washed and lysed in ice‐cold RIPA buffer (P0013J, Beyotime, China) supplemented with protease and phosphatase inhibitor cocktail (P1048, Beyotime, China) for 20 min, centrifuged, and pelleted at 15 000 × *g* at 4°C for 15 min. The supernatant was collected and quantified using the BCA Protein Assay Kit (P0012, Beyotime, China) at 562 nm absorbance on ELx800 ELIASA (BioTek, USA). The quantitative protein solution was diluted and boiled with 5× sodium dodecyl sulfate‐polyacrylamide gel electrophoresis (SDS‐PAGE) protein loading buffer (G2075, Servicebio, China) for 10 min. It was then separated on 10% SDS‐PAGE gels (PG112, Epozyme Biotech, China) by electrophoresis and transferred to Pierce polyvinylidene fluoride (PVDF) transfer membranes (88518, ThermoScientific, USA) using a Trans‐Blot Turbo Transfer System (BioRad). The PVDF membranes were blocked with 5% nonfat milk (P0216, Beyotime, China) supplemented with Tris‐buffered saline (TBS, G0001, Servicebio, China) and 0.1% Tween 20 (GC204002, Servicebio, China) for 1 h at room temperature. PVDF membranes were probed with different antibodies (SAT1, 1:800, Abcam, UK; ASS1, 1:800, Proteintech, USA; 3× Flag, 1:1000, Proteintech, USA; HA, 1:1000, Proteintech, USA; GAPDH, 1:3000, Proteintech, USA) at 4°C overnight. The membranes were rinsed with tris‐buffered saline Tween‐20 (TBST) the next day before being incubated with horseradish peroxidase‐conjugated secondary antibodies (anti‐rabbit IgG, 1:5000, Cell Signaling Technology, USA; anti‐mouse IgG, 1:5000, Cell Signaling Technology, USA) at room temperature for 1 h. Blots were visualized using the electrochemiluminescence (ECL) Western blotting substrate (32209, ThermoScientific, USA) on an Odyssey CLx Imager (Li‐Cor GmbH, Homburg, Germany).

### 5.21 Dual‐Luciferase Reporter Assay

The human SAT1 promoter was cloned into pGL3‐Basic plasmid (carrying luciferase reporter gene, supplied by Tsingke Biotech Co. (Beijing, China)). The sequence was shown in Table S11. MDA‐MB‐231 cells were plated in in vitro collagen density model at a density of 1 × 10^5^ cells each 24‐well plate. Subsequently, the cells were transfected with 0.5 µg per well of luciferase reporter plasmids. In addition, 10 ng of pRL‐TK plasmid (carrying the Renilla luciferase gene, from Tsingke Biotech Co.) was co‐transfected to serve as a normalizing control. After 48 h had elapsed post‐transfection, the luciferase activity was measured using the Dual‐Luciferase Reporter Assay System Kit (RG027, Beyotime, China), strictly adhering to the protocol provided by the manufacturer.

### 5.22 Protein–Protein Docking

Protein–protein docking was carried out with the GRAMM (Global Range Molecular Matching) web server (https://gramm.compbio.ku.edu/) to generate candidate complex structures [60].

### 5.23 IF Analysis

Cells or tissues were fixed with 4% paraformaldehyde (G1101, Servicebio, China) for 10 min and permeabilized with 0.5% Triton‐X100 (GC204003, Servicebio, China) in PBS for 5 min. Samples were rinsed with PBS, blocked with 5% BSA (GC305006, Servicebio, China) for 1 h at room temperature, and then incubated with primary antibodies (SAT1, 1:300, Abcam, UK; ASS1, 1:200, Proteintech, USA, palladin, 1:500, Abcam, UK) at 4°C overnight. The next day, samples were rewashed and incubated with a fluorescent dye‐conjugated secondary antibody and DAPI. Finally, the samples were washed and covered with an antifade mounting medium media (G1401, Servicebio, China) for microscopy. The images were acquired using an FV3000 Laser scanning confocal microscope (Olympus, Japan).

### 5.24 Lentivirus Packaging

The knockdown and overexpression of target genes and corresponding NC in the PLVX‐puro lentiviral vector were constructed by Tsingke Biotech Co. Lentiviral packaging mixtures were prepared by mixing 3,000 µL Opti–minimum essential medium (MEM, 31985070, Gibco, USA), 60 µL of Hieff Trans Liposomal Transfection Reagent (Yeasen, China), and 24 µg of plasmid mixture, which contained 12 µg of target plasmid, 3 µg of pMD2.G, and 9 µg of psPAX2. For lentivirus production, HEK293T cells at 70%–95% confluence were cultured in 10 mL of fresh Opti‐MEM with the above‐mentioned lentiviral packaging mixtures for 4–6 h. The supernatant was collected at 48 and 72 h post‐transfection, filtered through 0.22 µm syringe filters (FMC201030, BIOFIL, China), and incubated with target cells in the presence of 2 µg mL^−1^ polybrene (G4017, Servicebio, China) to construct stable silencing and overexpressing cell lines. Target sequences are listed in Table S12.

### 5.25 Measurement of Glu and Gln

The relative Glu/Gln concentrations of cells and tissues were detected using the glu colorimetric assay kit (E‐BC‐K903‐M, Elabscience Biotechnology, China) and gln colorimetric assay kit (E‐BC‐K853‐M, Elabscience Biotechnology, China), respectively, according to the manufacturer's instructions. The experimental design was the same as described in the MDA assay. For Glu detection, cells were homogenized in 0.9% NaCl (200 µL) and centrifuged at 12 000 × *g*, 4°C, for 10 min. The supernatant was filtered using a 10 KD ultrafiltration tube (FTT110105, BIOFIL, China) to remove impurities. The test specimen (30 µL) and standard solution (30 µL) were pipetted into 96‐well microplates. Then, 130 µL buffer solution and 60 µL enzyme reagent were added to The above wells and incubated at 37°C for 20 min in the dark. The absorbance at 450 nm was detected using a microplate reader (BioRad, USA). For Gln detection, cells or tissues were homogenized in 0.9% NaCl and centrifuged at 10 000 × *g* for 15 min at 4°C. Supernatant was filtered using a 50 KD ultrafiltration tube (FTT150105, BIOFIL, China). Then, 30 µL of diluted enzyme reagent A (enzyme reagent A: buffer solution = 1:9 in volume) was added to the standard and measuring wells. The standard solution (50 µL) and test specimen (50 µL) were pipetted into the standard wells and measuring wells, respectively, and then incubated at 37°C for 20 min in the dark. The absorbance at 450 nm (A1) was detected after adding 140 µL reaction working fluid (enzyme reagent B: substrate: chromogenic agent = 8: 1192: 200 in volume). Absorbance at 450 nm (A2) was again detected after incubation at 37°C for 30 min.

### 5.26 Immunoprecipitation (IP)

To perform IP, the supernatant of protein lysates was collected, and 10 µL of Anti‐Flag Magnetic Beads (HY‐K0207, MedChemExpress, USA) or Anti‐HA Magnetic Beads (HY‐K0201, MedChemExpress, USA) were added along with incubation for 2 h, rotating at room temperature. The beads were washed three times with TBST, magnetically separated on a magnetic rack, and then resuspended and boiled with 50 µL of 1× SDS‐PAGE protein loading buffer (G2075, Servicebio, Wuhan, China) for 5 min. The supernatant was collected for SDS‐PAGE and immunoblotting after the beads were magnetically separated.

### 5.27 Animal Experiments

Mice were acquired from Shanghai Model Organisms (Shanghai, China), kept in specific‐pathogen‐free conditions, and had free access to standard water and food in accordance with the institutional animal care and use guidelines of the Animal Care Committee of Fudan University Shanghai Cancer Center (Approval No. FUSCC‐IACUC‐2023337, Shanghai, China). A total of 5 × 10^5^ 4T1 cells supplemented with 100 µL 2.5 mg mL^−1^ or 50 µg mL^−1^ type I collagen solution (Advanced BioMatrix, USA) were inoculated into the fourth mammary fat pad of 4–6‐week‐old female BALB/c mice to establish an in vivo collagen density model. To develop a humanized xenograft model, a total of 1 × 10^6^ MDA‐MB‐231 cells supplemented with 100 µL 2.5 mg mL^−1^ or 50 µg mL^−1^ type I collagen solution were injected into the fourth mammary fat pad of 4–6‐week‐old female BALB/c nude mice. One week later, 1 × 10^7^ human peripheral blood mononuclear cells (Hu‐PBMCs) extracted from healthy donors were transplanted into BALB/c nude mice. For the drug intervention experiment, mice were intraperitoneally injected with Fer‐1 (0.2 mg every other day), anti‐mouse PD‐1 (200 µg every 3 days; BE0146, BioXcell, USA), isotype control (200 µg every 3 days; BE0089, BioXcell, USA) or 1% DMSO after one week of transplantation. SWE was performed regularly. Bodyweight (g) and tumor volume (width^2^ × length/2 mm^3^) were monitored every other day for each mouse in each group. At the time of euthanasia, the photographs and size of tumors were recorded.

### 5.28 IHC

Formalin‐fixed and paraffin‐embedded 4 µm thin tissue sections were deparaffinized and rehydrated using a Leica Bond Max automated stainer (Leica Biosystems GmbH, Nussloch, Germany), following the manufacturer's procedure. Antigen retrieval was conducted with 10 mM citric acid buffer (pH 6.0, G1219, Servicebio, China) for 20 min. The primary antibodies (SAT1, 1:300, Abcam, UK; ASS1, 1:300, Proteintech, USA; type I collagen, 1:1000, Proteintech, USA) were incubated for 15 min at room temperature and detected using a DAB color development kit (G1212, Servicebio, China). The expression level of the target protein was scored semi‐quantitatively based on staining intensity and distribution using the immunoreactive score (IRS), which was calculated as [61]: IRS = staining intensity (SI) × percentage of positive cells (PP). SI was defined as negative, 0; weak, 1; moderate, 2; and strong, 3. PP was defined as negative, 0; 1%–20% positive cells, 1; 21%–50% positive cells, 2; 51%–100% positive cells, 3. Ten visual fields from different areas of each slice were chosen randomly for the IRS assessment, and the average IRS was calculated as the final value.

### 5.29 Enzyme‐Linked Immunosorbent Assay (ELISA) for Human/Mouse IFN‐γ

The relative IFN‐γ concentrations were detected using the Mouse IFN‐γ ELISA Kit (U96‐1475E, YOBIBIO Biotechnology, China) and Human IFN‐γ ELISA Kit (U96‐3660E, YOBIBIO Biotechnology, China), respectively, according to the manufacturer's instructions.

### 5.30 Isolation of TIF

TIF was collected from tumors as previously described [62], with some changes. Tumors were excised, weighed, and rinsed with PBS to clear surface blood before being blotted dry. Each sample was placed in a 20 µm pluriStrainer nested in a microcentrifuge tube and centrifuged at 100 × g, 4°C, for 30 min. The liquid at the lower layer of the pluriStrainer was the TIF.

### 5.31 Isolation of Tumor‐Infiltrating Lymphocytes (TILs)

Tumor tissues were split with scissors and enzymatically digested with 1 mg mL^−1^ collagenase IV (17104019, Gibco, USA), 100 U mL^−1^ DNase I (10104159001, Sigma–Aldrich, USA), and 0.2 mg mL^−1^ hyaluronidase (abs44063396, Absin, China) in DMEM for 1 h at 37°C. Cell suspensions were filtered through 70 µm filters (CSS013070, BIOFOL, China) to attain single‐cell suspensions. TILs were enriched by density gradient centrifugation. TILs were washed with ice‐cold PBS and resuspended in a flow cytometry buffer for further use.

### 5.32 T Cell Isolation, Activation, and Co‐Culturation

Murine CD8^+^ T cells were isolated from the spleens of BALB/c mice using a Mouse CD8 T cell Enrichment Kit (8804‐6825‐74, ThermoScientific, USA). Over 90% purity was confirmed by flow cytometry. The CD8^+^ T cells were suspended in RPMI 1640 medium supplemented with 10% FBS, 50 µM β‐mercaptoethanol, and 50 U mL^−1^ recombinant mouse IL‐2. Then, the cells were activated with anti‐CD3 (eBioscience, 5 µg mL^−1^) and CD28 (eBioscience, 2.5 µg mL^−1^) for 48 h.

Human CD8^+^ T cells were extracted from the peripheral blood of healthy donors using the Human CD8 T cell Enrichment Kit (8804‐6812‐74, ThermoScientific, USA). The isolated human CD8^+^ T cells were suspended in CTS OpTmizer T Cell Expansion Basal Medium (A3705001, ThermoScientific, USA) and activated with anti‐CD3 antibody (5 µg mL^−1^) for 48 h. According to FACS analysis, the purity of the T cells was over 90%.

The cancer cells were mixed with a suspension of activated CD8^+^ T cells in complete RPMI 1640 medium at a ratio of 10:1 and co‐cultured at 37°C for 5 h.

### 5.33 Flow Cytometry

For cytokine staining, cells were incubated in fresh medium supplemented with a leukocyte activation cocktail (550583, BD Pharmingen, USA) at 37°C for 4 h. The samples were stained with Live/Dead dye (Zombie Aqua Fixable Viability Kit, 423101, Biolegend) for 15 min at room temperature. After being labeled with antibodies against cell surface markers, cells were fixed and permeabilized using the relevant buffer set (420801, 421002, BioLegend, USA) for 20 min to disrupt the cell membrane. They were then treated with fluorochrome‐conjugated antibodies. The stained cells were run through a FACS flow cytometer (BD Biosciences, USA), and the resulting data were processed using CytExpert software.

The antibodies (BioLegend) used for the mouse TILs included APC/Cyanine7‐conjugated anti‐CD45 (103116), FITC‐conjugated anti‐CD3 (100306), APC‐conjugated anti‐CD4 (100515), perCP/Cyanine5.5‐conjugated anti‐CD8a (100734), Brilliant violet 605‐conjugated anti‐IL‐2 (503829), Brilliant violet 421‐conjugated anti‐TNF‐α (506327), PE‐conjugated anti‐IFN‐γ (505807), TruStain FcX anti‐CD16/32 (101320), PE‐conjugated anti‐CD19 (115507), PE/Cyanine7‐conjugated anti‐CD11b (101215), perCP/Cyanine5.5‐conjugated anti‐CD80 (104715), and APC‐conjugated anti‐CD163 (156705).

The antibodies (BioLegend) used for the human TILs included APC/Cyanine7‐conjugated anti‐CD45 (304014), FITC‐conjugated anti‐CD3 (317305), APC‐conjugated anti‐CD4 (300514), perCP/Cyanine5.5‐conjugated anti‐CD8a (300923), Brilliant violet 605‐conjugated anti‐IL‐2 (500331), Brilliant violet 421‐conjugated anti‐TNF‐α (376213), PE‐conjugated anti‐IFN‐γ (383303), Fc Receptor Blocking Solution (422302), PE‐conjugated anti‐CD19 (302207), PE/Cyanine7‐conjugated anti‐CD11b (340009), perCP/Cyanine5.5‐conjugated anti‐CD80 (375411), and APC‐conjugated anti‐CD163 (326509).

The gating strategy that was used to identify immune cell subsets was as follows: CD8^+^ T cells (CD45^+^ CD3^+^ CD8^+^); CD4^+^ T cells (CD45^+^ CD3^+^ CD4^+^); B cells (CD45^+^ CD3^−^ CD19^+^); macrophage M1 (CD45^+^ CD11b^+^ CD80^+^); and macrophage M2 (CD45^+^ CD11b^+^ CD163^+^). The detailed gating strategy was provided in Figure S13.

### 5.34 Survival Analysis

Survival and survminer packages in the R software (version 4.0.2) were used to perform the survival analysis. Kaplan–Meier plot and log‐rank tests were conducted to assess the associations between SAT1 expression and the survival of patients with breast cancer. A *p* < 0.05 was considered to be statistically significant.

### 5.35 Statistical Analysis

All experiments were performed in triplicate. In this study, Shapiro‐Wilk methods was used to test the data's normality. For comparisons between two groups, Student's t‐test (normally distributed) or Mann‐Whitney U test (non‐normally distributed) was used. For more than two groups, one‐way ANOVA (normally distributed) or Kruskal‐Wallis H test (non‐normally distributed) was used, followed by post‐hoc tests. All statistical analyses were performed using the GraphPad Prism (version 8.0) or R software (version 4.0.2). A *p* < 0.05 (two‐sided) was considered statistically significant. * indicated *p* < 0.05, ** indicated *p* < 0.01, and *** indicated *p* < 0.001.

## Author Contributions

**Jinyan Wang**: conceptualization, validation, formal analysis, supervision, methodology, data curation, funding acquisition, writing – original draft, writing – review and editing, project administration. **Shuangyue Pan**: conceptualization, data curation, investigation, validation, writing – review and editing, writing – original draft, formal analysis, supervision. **Lu Bai**: methodology, data curation. **Bin Li**: data curation. **Tiantian Liu**: data curation. **Jianing Cao**: data curation. **Mengdi Yang**: data curation. **Heda Zhang**: data curation. **Duancheng Guo**: data curation. **Wenxiang Zhi**: data curation, methodology. **Zhonghua Tao**: conceptualization, writing – review and editing, methodology, supervision. **Xichun Hu**: conceptualization, writing – review and editing, funding acquisition, methodology, investigation, supervision, project administration.

## Conflicts of Interest

The authors declare no conflicts of interest.

## Supporting information




**Supporting File 1**: advs77879‐sup‐0001‐SuppMat.docx.

**Supporting File 2**: advs77879‐sup‐0002‐SuppMat.xlsx.

## Data Availability

The data that support the findings of this study are openly available in the National Center for Biotechnology Information (NCBI) at https://www.ncbi.nlm.nih.gov/geo/query/acc.cgi?acc=GSE284280, reference number GSE284280.
