# Supplementary material for: High‐Density Type I Collagen Promotes IFN‐γ+ CD8+ T Cell Exhaustion via SAT1‐ASS1‐Mediated Glutamine Accumulation and Ferroptosis in Triple‐Negative Breast Cancer
Source: Adv Sci (Weinh). 2026 Sep 27:e77879. Online ahead of print. doi: 10.1002/advs.77879 (PMC13616355; doi:10.1002/advs.77879)
Supplement: Supplementary file 1 [file ADVS-9999-e77879-s002.docx]

**Supplementary Figures**

**Figure. S1. The establishment and verification of the *in vitro*** **collagen density model.** Shear moduli of different type I collagen solutions (high density, 2.5 mg mL^-1^, (a); modest density, 1 mg mL^-1^, (b); low density, 50 µg mL^-1^, (c) during liquid-to-solid gelation over 60 min, represented as storage modulus (G’) and loss modulus (G’’). (d) The tanδ of the different *in vitro* collagen density model at 60 min. *n* = 5. (e) The stress relaxation curve of the different *in vitro* collagen density model. (f) The normolized stress relaxation rate of the different *in vitro* collagen density model. *n* = 5. (g-i) Shear moduli (G’ and G’’) of pre-formed collagen substrates from 0.1 to 10 Hz over a logarithmic scale. Statistical significance was determined by two-tailed unpaired t-test (d, f).

**Figure. S2. Collagen density induces the alteration of actin fiber of TNBC cells.** The actin fiber morphology of BT-549 and 4T1 cells cultured on type I collagen at different densities. Blue indicated DAPI, and Red indicated actin fiber.

**F****igure. S3.** **The apoptosis and necrosis rate of TNBC cells under different collagen densities.** (a) The apoptosis and necrosis rate of MDA-MB-231 cells cultured on type I collagen at different densities. (b) The apoptosis and necrosis rate of BT-549 cells cultured on type I collagen at different densities. (c) The apoptosis and necrosis rate of 4T1 cells cultured on type I collagen at different densities. Statistical significance was determined by two-tailed unpaired t-test (a-c). Unless otherwise specified, *n* = 3. All studies were performed with three biological replicates. Values are shown as the means ± SDs.

**Figure.** **S4. The** **proliferation rate of TNBC cells cultured on type I collagen at different densities.** (a) The proliferation rate of MDA-MB-231 cells cultured on type I collagen at different densities. (b) The proliferation rate of BT-549 cells cultured on type I collagen at different densities. (c) The proliferation rate of 4T1 cells cultured on type I collagen at different densities. Statistical significance was determined by two-tailed unpaired t-test (a-c). Unless otherwise specified, *n* = 3. All studies were performed with three biological replicates. Values are shown as the means ± SDs.

**Figure. S5. The** **colony formation of TNBC cells cultured on type I collagen at different densities.** (a) The colony formation of MDA-MB-231 cells cultured on type I collagen at different densities. (b) The colony formation of BT-549 cells cultured on type I collagen at different densities. (c) The colony formation of 4T1 cells cultured on type I collagen at different densities**.**

**Figure. S6: Mitochondrial morphology and membrane potential** **of TNBC cells cultured on type I collagen at different densities.** (a) Mitochondrial morphology of BT-549 and 4T1 cultured on type I collagen at different densities. (b) The mitochondrial membrane potential of BT-549 and 4T1 cultured on type I collagen at different densities was measured by using the JC-1 probe.

**Figure. S7: IF analysis of SAT1 in TNBC cells and the establishment of SAT1-silenced/ overexpressed TNBC cell lines.** IF analysis of SAT1 (in green) and palladin (in red) in BT-549 (a) and 4T1 (b), cultured on type I collagen at different densities. RT-qPCR and immunoblotting analysis of SAT1 in SAT1-silenced MDA-MB-231 (c), BT-549 (d), and 4T1 (e), compared with the NC. RT-qPCR and immunoblotting analysis of SAT1 in SAT1-overexpressed MDA-MB-231 (f), BT-549 (g), and 4T1 (h), compared with the NC. Statistical significance was determined by two-tailed unpaired t-test (c-h). Unless otherwise specified, *n* = 3. All studies were performed with three biological replicates. Values are shown as the means ± SDs.

**Figure. S8. The ferroptosis level and Gln accumulation in the *in vitro*** **collagen density model of SAT1-silenced/overexpressed TNBC cells.** (a) Lipid formation, Fe^2+^, and Gln levels were examined in the *in vitro* collagen density model of the indicated TNBC cells. Immunoblotting analysis of SAT1 in 3’UTR-SAT1-silenced (sh-SAT1-3) MDA-MB-231 (b), BT-549 (c), and 4T1 (d), compared with the NC. Immunoblotting analysis of SAT1 in SAT1-overexpressed MDA-MB-231 (e), BT-549 (f), and 4T1 (g), which were pre-silenced of 3’UTR-SAT1, compared with the NC. (h) Lipid formation, Fe^2+^, and Gln levels were examined in the indicated TNBC cells. Statistical significance was determined by two-tailed unpaired t-test (a, h). Unless otherwise specified, *n* = 3. All studies were performed with three biological replicates. Values are shown as the means ± SDs.

**Figure. S9: The establishment of ASS1-silenced/overexpressed TNBC cells and the ferroptosis level in ASS1-silenced/overexpressed TNBC cells.** Immunoblotting analysis of ASS1 in ASS1-silenced MDA-MB-231 (a), BT-549 (b) and 4T1 (c), compared with the NC. Immunoblotting analysis of ASS1 in ASS1-overexpressed MDA-MB-231 (d), BT-549 (e), and 4T1 (f), compared with the NC. (g) Lipid formation, Fe^2+^, and Gln levels were examined in the *in vitro* collagen density model of the indicated cells (OE-ASS1 and OE-NC). (h) Lipid formation, Fe^2+^, and Gln levels were examined in the indicated cells. (i) Lipid formation, Fe^2+^, and Gln levels were examined in the indicated cells. Lipid formation (j), Fe^2+^ (k), and Gln (l) levels were examined in the indicated cells. Lipid formation (m), Fe^2+^ (n), and Gln (o) levels were examined in the indicated cells. Statistical significance was determined by two-tailed unpaired t-test (g-o). Unless otherwise specified, *n* = 3. All studies were performed with three biological replicates. Values are shown as the means ± SDs.

**Figure. S10. High-density collagen promotes Gln accumulation–related ferroptosis and IFN-γ^+^ CD8^+^ T cells exhaustion *in vivo*.** (a) The SWE Emean values of tumors during the establishment process of *in vivo* collagen density model of MDA-MB-231. *n* = 5. (b) The image of SWE Emean value of tumors in *in vivo* collagen density model of MDA-MB-231 at day 21. The loss (c), storage (d) modulus, tan δ (e) and stress relaxation (f) of tumors detected by rheometer in *in vivo* collagen density model of MDA-MB-231 at day 21. *n* = 5. (g) The image of tumors derived from MDA-MB-231 in *in vivo* collagen density model. *n* = 5. (h) The growth curve of tumors in *in vivo* collagen density model of MDA-MB-231. *n* = 5. Lipid formation (i), Fe^2+^ (j), Gln (k) and TIF Gln (l) levels were examined in tumors of *in vivo* collagen density model of MDA-MB-231. *n* = 5. (m) IHC images of SAT1 and ASS1 in *in vivo* collagen density model of MDA-MB-231. The proportion of CD4^+^ and CD8^+^ T cells in TME of *in vivo* collagen density model of 4T1 (n) and MDA-MB-231 (o). (p) The proportion of TILs (B cells, macrophage M1, macrophage M2, CD4^+^, and CD8^+^ T cells) in TME of *in vivo* collagen density model of MDA-MB-231. *n* = 5. (q) The proportion of intracellular cytokines in CD8^+^ T cells of *in vivo* collagen density model of MDA-MB-231. *n* = 5. The proportion of IFN-γ in CD8^+^ T cells of *in vivo* collagen density model of 4T1 (r) and MDA-MB-231 (s). Statistical significance was determined by two-tailed unpaired t-test (a,c-f, i-l, p-q) and two way analysis of variance (ANOVA) (h). Unless otherwise specified, *n* = 3. All studies were performed with three biological replicates. Values are shown as the means ± SDs.

**Figure. S11:** **The co-culturation of TNBC and CD8^+^ T cells.** (a) The co-culturation pattern of TNBC and CD8^+^ T cells in *in vitro* collagen density model. (b) Gln levels in media were examined in the co-culturation between TNBC and CD8^+^ T cells. (c) IFN-γ levels in media were examined in the co-culturation between TNBC and CD8^+^ T cells. (d) The viability of TNBC cells after co-culturation with CD8^+^ T cells. (e) IFN-γ levels in media were examined through culturing CD8⁺ T cells in Gln-free, Gln-replete or Gln-excess medium. (f) The co-culturation pattern of TNBC and CD8^+^ T cells in which SAT1 was stably silenced or overexpressed. (g-h) Gln levels in TNBC cells and media through co-culturing CD8⁺ T cells and TNBC cells in which SAT1 was stably silenced. (i) IFN-γ levels in media through co-culturing CD8⁺ T cells and TNBC cells in which SAT1 was stably silenced. (j-k) Gln levels in TNBC cells and media through co-culturing CD8⁺ T cells and TNBC cells in which SAT1 was stably overexpressed. (l) IFN-γ levels in media through co-culturing CD8⁺ T cells and TNBC cells in which SAT1 was stably overexpressed. Statistical significance was determined by two-tailed unpaired t-test (b-e, g-l). Unless otherwise specified, *n* = 3. All studies were performed with three biological replicates. Values are shown as the means ± SDs.

**Figure. S12: The proportion of TILs in the TME of the *in vivo* collagen density model of 4T1 treated with Fer-1 or DMSO.** (a) The viability of CD8+ T cells cultured in Fer-1-containing medium or standard medium. (b) The proportion of CD4^+^ and CD8^+^ T cells in the TME of the *in vivo* collagen density model of 4T1 treated with Fer-1 or DMSO. (c) The proportion of IFN-γ in CD8^+^ T cells in TME of the *in vivo* collagen density model of 4T1 treated with Fer-1 or DMSO. Statistical significance was determined by two-tailed unpaired t-test (a). All studies were performed with three biological replicates. Values are shown as the means ± SDs.

**Figure. S13: Detailed gating strategy of flow cytometry.**
